# TRIM55 restricts the progression of hepatocellular carcinoma through ubiquitin-proteasome-mediated degradation of NF90

**DOI:** 10.1038/s41420-024-02212-y

**Published:** 2024-10-17

**Authors:** Changhong Luo, Yuyan Lu, Qinliang Fang, Jing Lu, Ping Zhan, Wenqing Xi, Jinzhu Wang, Xijun Chen, Qin Yao, Fuqiang Wang, Zhenyu Yin, Chengrong Xie

**Affiliations:** 1 Fujian Provincial Key Laboratory of Chronic Liver Disease and Hepatocellular Carcinoma, Xiamen Translational Medical Key Laboratory of Digestive System Tumor, Zhongshan Hospital of Xiamen University, Xiamen, Fujian Province China; 2 Department of Hepatobiliary Surgery, Xiamen Key Laboratory of Liver Diseases, Xiamen Hospital of Traditional Chinese Medicine, Beijing University of Chinese Medicine, Xiamen, Fujian Province China; 3 College of Integrative Medicine, Fujian University of Traditional Chinese Medicine, Fuzhou, Fujian Province China; 4 Central Laboratory, Zhongshan Hospital of Xiamen University, School of Medicine, Xiamen University, Xiamen, Fujian Province China

**Keywords:** Liver cancer, Ubiquitylation

## Abstract

Hepatocellular carcinoma (HCC) is a prevalent malignant tumor worldwide. Tripartite motif containing 55 (TRIM55), also known as muscle-specific ring finger 2 (Murf2), belongs to the TRIM protein family and serves as an E3 ligase. Recently, the function and mechanism of TRIM55 in the advancement of solid tumors have been elucidated. However, the role of TRIM55 and its corresponding protein substrates in HCC remains incompletely explored. In this study, we observed a significant reduction in TRIM55 expression in HCC tissues. The downregulation of TRIM55 expression correlated with larger tumor size and elevated serum alpha-fetoprotein (AFP), and predicted unfavorable overall and tumor-free survival. Functional experiments demonstrated that TRIM55 suppressed the proliferation, migration, and invasion of HCC cells in vitro, as well as hindered HCC growth and metastasis in vivo. Additionally, TRIM55 exhibited a suppressive effect on HCC angiogenesis. Mechanistically, TRIM55 interacted with nuclear factor 90 (NF90), a double-stranded RNA-binding protein responsible for regulating mRNA stability and gene transcription, thereby facilitating its degradation via the ubiquitin-proteasome pathway. Furthermore, TRIM55 attenuated the association between NF90 and the mRNA of HIF1α and TGF-β2, consequently reducing their stability and inactivating the HIF1α/VEGF and TGFβ/Smad signaling pathways. In conclusion, our findings unveil the important roles of TRIM55 in suppressing the progression of HCC partly by promoting the degradation of NF90 and subsequently modulating its downstream pathways, including HIF1α/VEGF and TGFβ/Smad signaling.

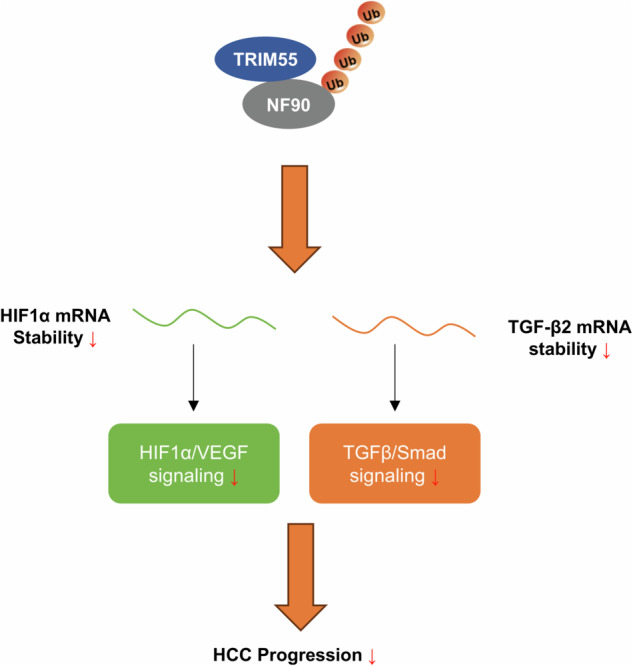

## Introduction

Hepatocellular carcinoma (HCC) is a prevalent malignancy that affects people worldwide, which ranks as the third leading cause of cancer-related mortality. This persistent increase in HCC cases poses a significant challenge to global health, as treatment options remain limited [1, 2]. Emanating from diverse etiologies such as metabolic-associated fatty liver disease, alcoholic liver disease, autoimmune hepatitis, and chronic hepatitis B or hepatitis C infection, HCC emerges as a consequence of genetic and epigenetic alterations, regulation of oxidative stress, modulation of inflammatory responses, and active participation of the immune system [3–5]. New evidence from diverse model systems suggests that a limited subset of cellular signaling pathways may be sequentially implicated in the process of hepatocarcinogenesis and the progression of tumors with metastasis or recurrence [6]. However, it is important to note that our comprehension of the modified signaling pathways in HCC remains incomplete.

Tripartite motif containing 55 (TRIM55), also known as muscle-specific ring finger 2 (Murf2), is a member of TRIM protein family and functions as a E3 ligase. TRIM55 plays a crucial role in the early stages of skeletal muscle differentiation and myofiber formation [7]. It is associated with conditions such as myocardial hypertrophy and heart failure [8, 9]. Recently, the function and mechanism of TRIM55 in progression of solid tumors has been reported. For example, downregulation of TRIM55 is closely associated with chemo-resistance, migration, and cancer stem-cell-like phenotype of lung adenocarcinoma cells via regulation of Snail1 degradation [10]. In colorectal cancer, TRIM55 induces the degradation of oncoprotein c-Myc to suppress growth and metastasis [11]. Conversely, TRIM55 expression is significantly increased in gastric cancer tissues and functions as an oncogene to promote epithelial-mesenchymal transition (EMT) and metastasis [12]. However, the comprehensive exploration of the TRIM55-mediated ubiquitination of its corresponding protein substrates and its functional significance in HCC remains incomplete.

Nuclear factor 90 (NF90), encoded by *interleukin enhancer-binding factor-3* (*ILF3*), is a double-stranded RNA-binding protein that forms a complex with NF45 to regulate mRNA stability and gene transcription, playing crucial roles in RNA metabolism, processing of pri-miRNAs, viral replication, and mitosis [13–16]. NF90 is upregulated in various tumors, and its target mRNAs include HIF1α, PARP1, cyclin E1, TMEM98, VEGF, among others, suppressing the degradation of these target RNAs and inducing tumor proliferation, invasion, and angiogenesis [16–21]. Ubiquitination is a reversible post-translational modification that regulates various biological functions such as gene transcription, DNA damage repair, proliferation, and motility, by influencing protein folding, localization, degradation, and activity [22]. Recently, the regulation of ubiquitin on NF90 has been preliminarily investigated. In macrophages, E3 ubiquitin ligase TRIM47 associates with NF90 and initiates a proteasome-dependent degradation, which is crucial for the antiviral innate immunity [23]. Our previous research has demonstrated that the deubiquitinase USP11 binds to NF90, resulting in its deubiquitination and stabilization. This, in turn, promotes the proliferation and metastasis of HCC cells [24]. A cytosolic yeast analysis conducted in a previous study has identified a potential association between TRIM55 and NF90 [25]. However, to date, the regulatory relationship between TRIM55 and NF90 remains unclear. In this study, we have shown that reduced expression of TRIM55 predicts an unfavorable clinical outcome for patients with HCC. Furthermore, we investigated the functional relevance between TRIM55 and NF90, as well as their downstream signaling pathways.

## Results

### Reduced expression of TRIM55 is correlated with unfavorable clinical prognosis of HCC patients

**Fig. 1:**
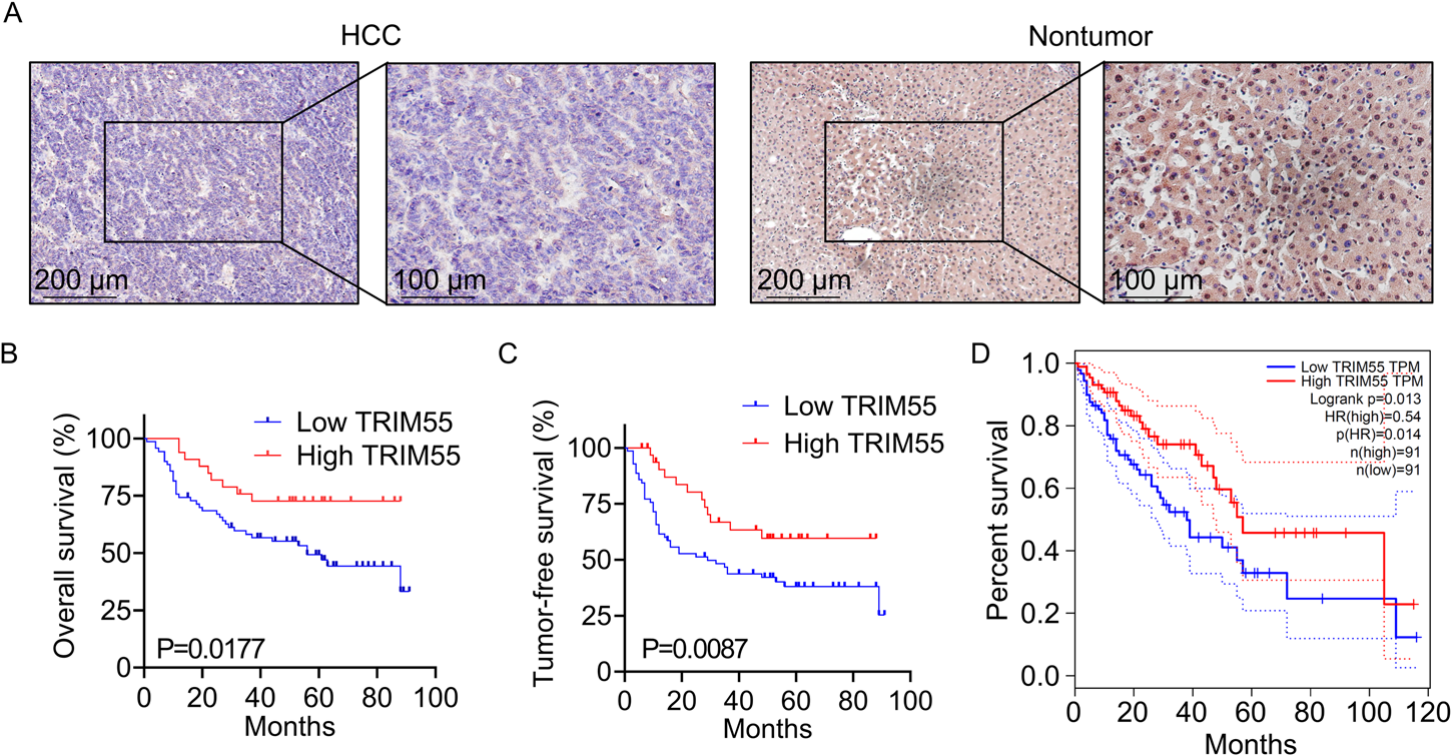
Downregulation of TRIM55 predicts poor clinical outcome in patients with HCC. **A** The expression of TRIM55 in 103 pairs of HCC and corresponding adjacent nontumor tissues was examined by the immunohistochemistry (IHC) assay. The representative images were shown. **B**, **C** Kaplan–Meier survival analysis of the overall survival (**B**) and tumor-free survival (**C**) in the HCC patients based on TRIM55 expression. The low expression patients (n = 72) showed an expression value of HCC tissues less than the corresponding adjacent nontumor tissues, while the high expression patients (n = 31) showed the expression value of HCC tissues larger than the corresponding adjacent nontumor tissues. **D** The TCGA database analysis on the association between TRIM55 expression and overall survival.

To validate the expression pattern of TRIM55 in HCC, the immunohistochemical (IHC) staining method was carried out to examine the levels of TRIM55 protein in 103 pairs of HCC and corresponding adjacent nontumor tissues. As shown in Fig. 1A, a notable 70% (72/103) of HCC tissues expressed significantly lower levels of TRIM55 compared to the adjacent nontumor tissues. To determine the clinical significance of TRIM55 in HCC patients, the association between TRIM55 levels and clinicopathological features was analyzed. Intriguingly, we discovered a significant association between reduced TRIM55 expression and larger tumor size (*p* = 0.008) as well as elevated serum alpha-fetoprotein (AFP) levels (*p* = 0.026). However, no conspicuous correlation was observed between TRIM55 expression and other factors, including age, gender, differentiation grade, satellite lesion, vascular thrombus, serum HBV level, cirrhosis, and metastasis (Table 1). Moreover, the Kaplan–Meier survival analysis revealed that HCC patients with low TRIM55 expression experienced significantly shorter overall and tumor-free survival times compared to those with high TRIM55 expression (Fig. 1B, C). Furthermore, the analysis of The Cancer Genome Atlas (TCGA) data utilizing the GEPIA online tool further substantiated our findings (Fig. 1D). Collectively, these data strongly suggest that the downregulation of TRIM55 is intricately linked to the malignant progression of HCC.

**Table 1:** The correlation between TRIM55 expression and clinicopathological features of HCC patients.

Clinicopathological features	Low TRIM55	High TRIM55	*P* value
Age			
<60	43 (41.7%)	18 (17.5%)	0.875
≥60	29 (28.2%)	13 (12.6%)	
Gender			
Male	58 (56.3%)	27 (26.2%)	0.423
Female	14 (13.6%)	4 (3.9%)	
Tumor size			
≤5 cm	24 (23.3%)	19 (18.4%)	0.008
>5 cm	48 (46.6%)	12 (11.7%)	
Differentiation level			
Low/Medium	8 (7.8%)	1 (1.0%)	0.194
High	64 (62.1%)	30 (29.1%)	
Satellite focus			
Without	57 (55.3%)	26 (25.2%)	0.579
With	15 (14.6%)	5 (4.9%)	
Serum AFP (ng/mL)			
<200	39 (37.9%)	24 (23.3%)	0.026
≥200	33 (32%)	7 (6.8%)	
PVTT			
Without	41 (35.9%)	17 (17.5%)	0.843
With	31 (34.0%)	14 (12.6%)	
Serum HBV (IU/mL)^a^			
<1000	29 (36.9%)	18 (19.4%)	0.270
≥1000	31 (33.0%)	13 (10.7%)	
Liver cirrhosis status			
No	46 (44.7%)	18 (17.5%)	0.576
Yes	26 (25.2%)	13 (12.6%)	
Metastasis			
Without	51 (50.5%)	25 (25.2%)	0.2990
With	21 (19.4%)	6 (4.9%)	

*AFP* Alpha-fetoprotein, *PVTT* Portal vein tumor thrombus, *HBV* Hepatitis B virus.

^a^Twelve missing data points.

### TRIM55 suppresses the malignant phenotypes of HCC cells

**Fig. 2:**
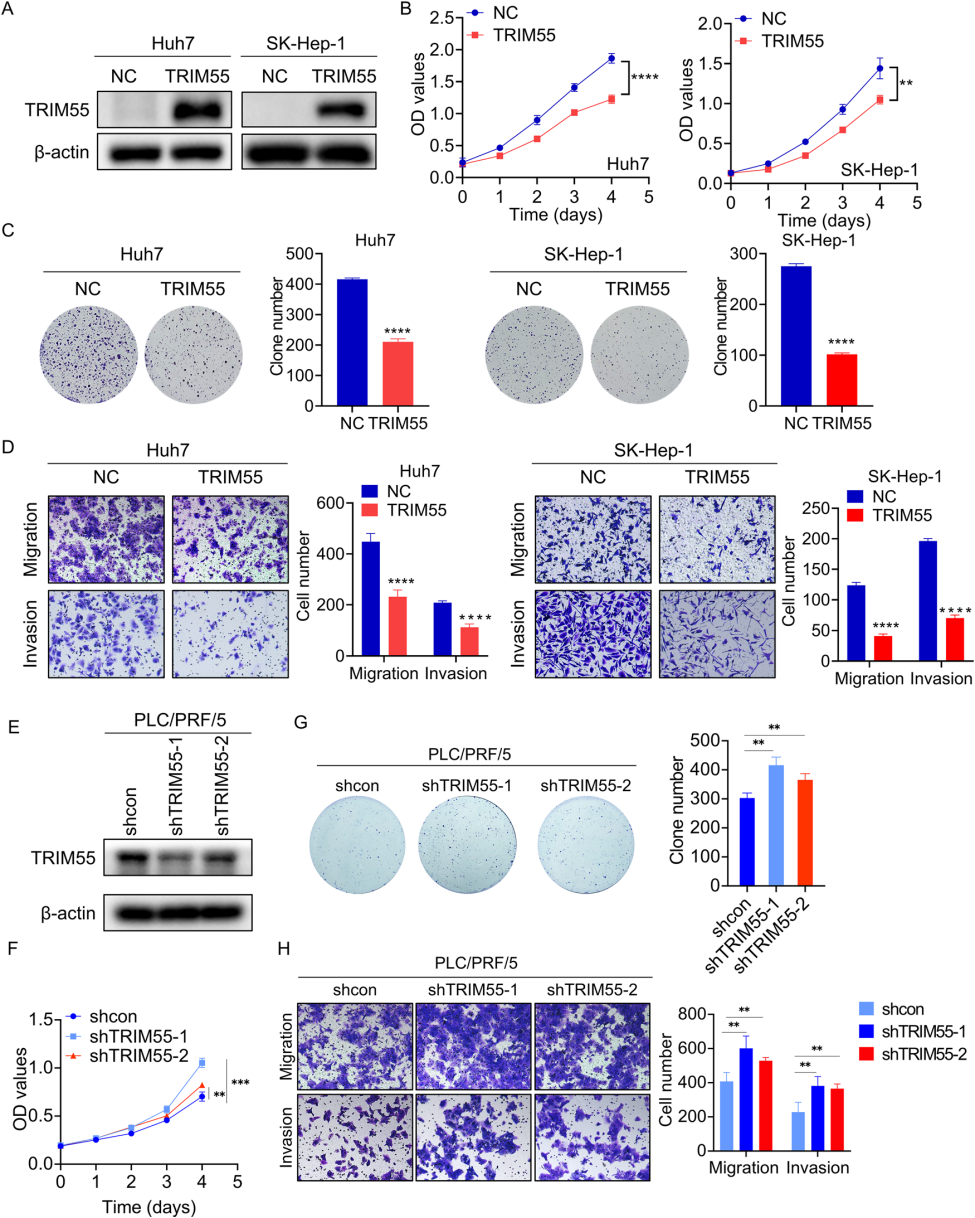
TRIM55 inhibits the malignant phenotypes of HCC cells. **A** TRIM55 expression level in Huh7 and SK-Hep-1 cells infected with lentivirus expressing vector and TRIM55. **B** CCK-8 assays were used to determine the proliferation of Huh7 and SK-Hep-1 after TRIM55 overexpression. **C** Colony formation assays for HCC cells with or without TRIM55 overexpression. Colonies were counted and imaged. **D** The representative images of transwell assay for the control and TRIM55 overexpressing Huh7 and SK-hep-1 cells. The cells were counted under a microscope in five randomly selected fields. **E** TRIM55 expression level in PLC/PRF/5 cells infected with lentivirus expressing vector and TRIM55 shRNA. **F** CCK-8 assays were used to determine the proliferation of PLC/PRF/5 after TRIM55 silence. **G** Colony formation assays for PLC/PRF/5 cells with or without TRIM55 knockdown. Colonies were counted and imaged. **H** The representative images of transwell assay for the control and TRIM55 knockdown PLC/PRF/5 cells. The cells were counted under a microscope in five randomly selected fields. *n* = 3, **P < 0.01, ***P < 0.001, ****P < 0.0001.

To evaluate the functional significance of TRIM55 in HCC progression, we conducted overexpression experiments in Huh7 and SK-Hep-1 cells (Fig. 2A). Through CCK-8 and colony formation assays, it was observed that the proliferation capacity was significantly diminished upon TRIM55 overexpression, as compared to the control cells (Fig. 2B, C). Furthermore, transwell assays revealed a notable reduction in migratory and invasive abilities of both Huh7 and SK-Hep-1 cells with TRIM55 overexpression, in comparison to the control group (Fig. 2D). Conversely, TRIM55 was knockdown in PLC/PRF/5 which expressed higher TRIM55 (Fig. 2E). It was demonstrated that depletion of TRIM55 expression significantly led to a promotion of cellular proliferation, migration and invasion in PLC/PRF/5 cells (Fig. 2F–H).

**Fig. 3:**
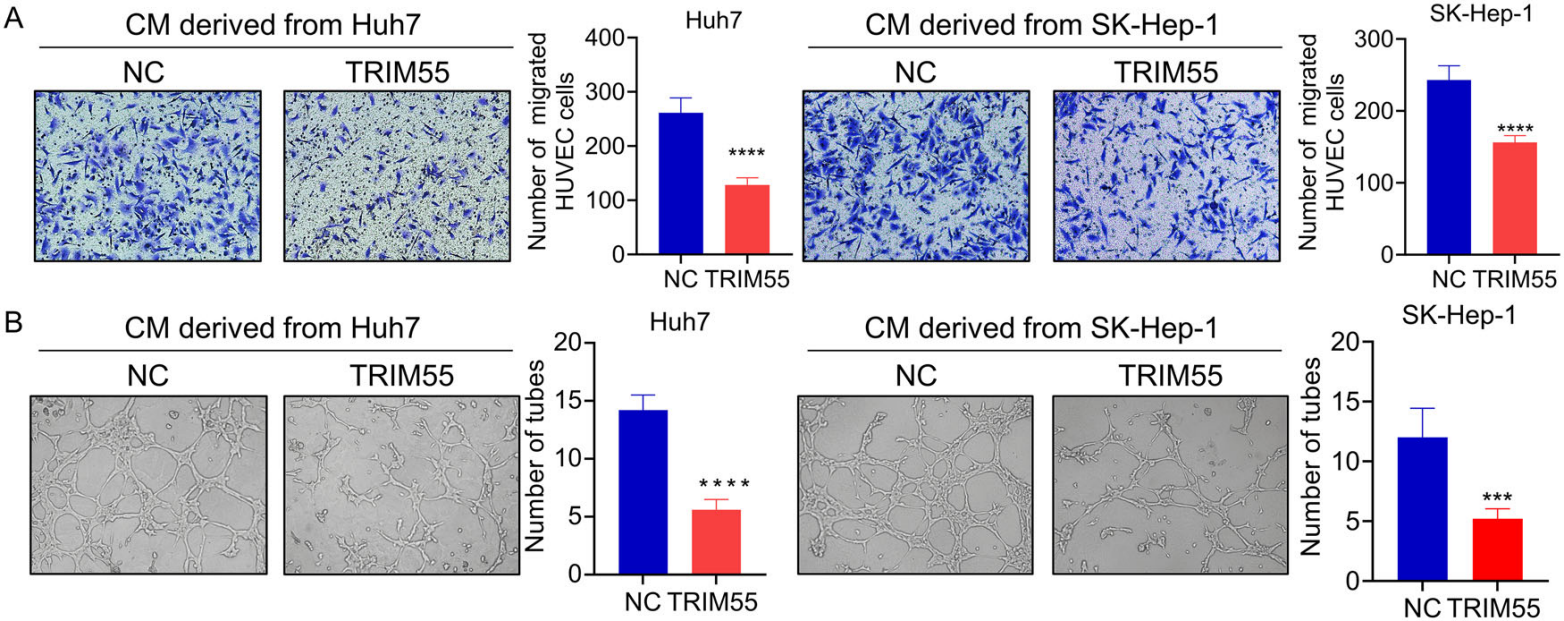
TRIM55 inhibits HCC angiogenesis. **A** Transwell assay testing the HUVECs migration affected by the CM derived from control and TRIM55-overexpressed HCC cells. **B** HUVECs tube formation affected by the CM derived from control and TRIM55-overexpressed HCC cells. *n* = 3, ***P < 0.001, ****P < 0.0001.

Given that angiogenesis serves as the foundation for tumor growth, invasion, and metastasis, we sought to explore the functional significance of TRIM55 in the regulation of HCC angiogenesis. Following a 48-h incubation period, the conditioned mediums (CM) from both control and TRIM55-overexpressed HCC cells were collected and subsequently applied to culture human umbilical vein endothelial cells (HUVECs) for an additional 48 h. Through the implementation of transwell and tube-formation experiments, it became evident that the CM derived from TRIM55-overexpressed HCC cells significantly impeded the migration and tube formation of HUVECs in comparison to that obtained from the control group (Fig. 3A, B). Collectively, our findings suggest that TRIM55 inhibits the malignant behaviors of HCC cells in vitro.

### TRIM55 represses the HCC progression in vivo

**Fig. 4:**
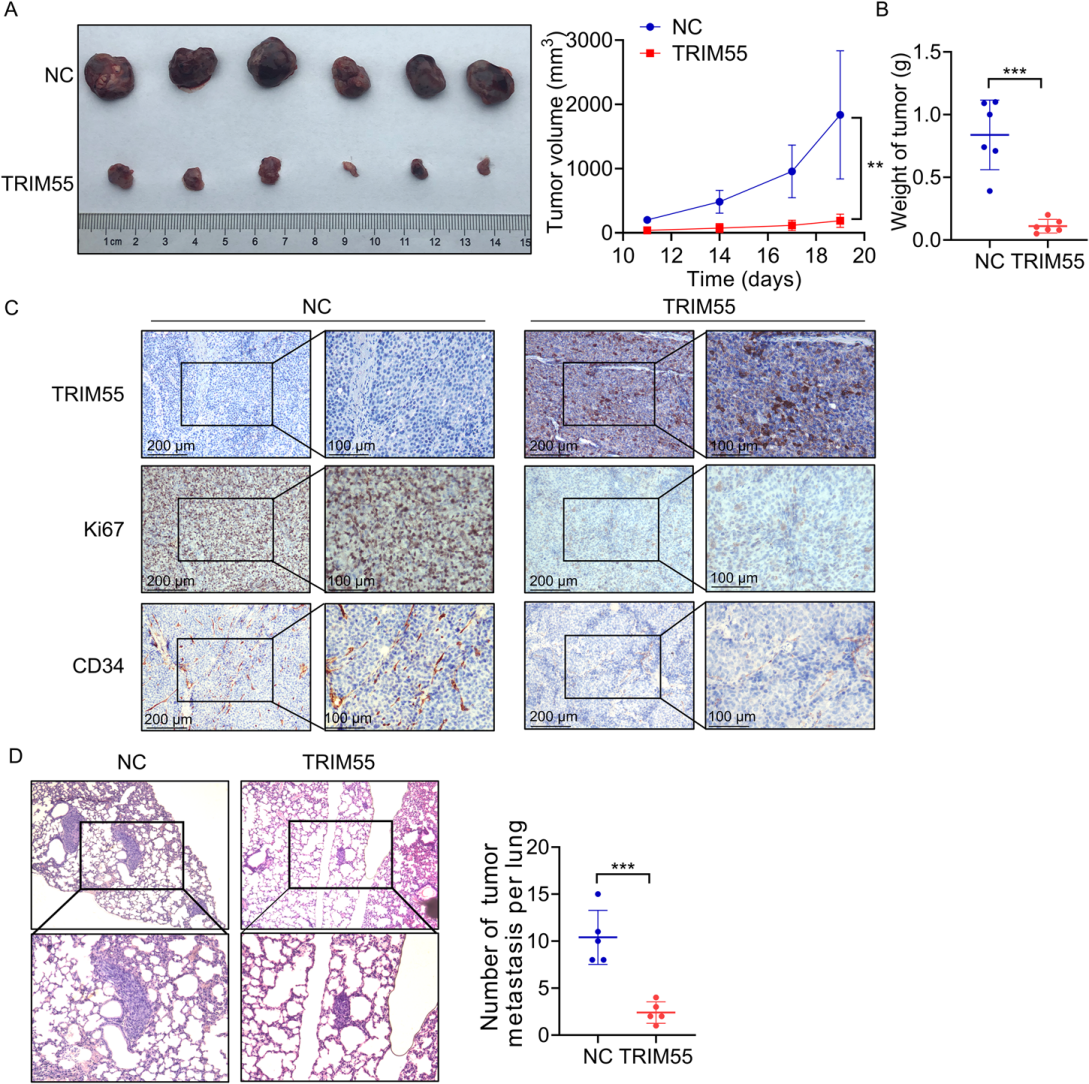
TRIM55 inhibits HCC progression in vivo. **A** Huh7 cells with stable TRIM55 overexpression were injected into nude mice. Tumor volumes were monitored every 3 days after injection. **B** The tumor weight of xenograft from the different groups was calculated. **C** IHC staining of TRIM55, Ki67, and CD34 in tumor tissue from nude mice. **D** Huh7 cells with stable TRIM55 overexpression were injected into the tail vein of nude mice. 8 weeks later, the pulmonary nodules were examined by H&E staining. The represent images and statistical result were shown. **P < 0.01, ***P < 0.001.

To further validate the anti-proliferative effect of TRIM55 in vivo, we subcutaneously injected control and TRIM55-overexpressed Huh7 cells into nude mice. Tumors derived from TRIM55-overexpressed Huh7 cells exhibited considerably slower growth compared to those originating from control cells (Fig. 4A). Moreover, both tumor weight and volume were significantly lower in the TRIM55 overexpression group than in the control group (Fig. 4A, B). Additionally, the IHC analysis revealed a marked decrease in the positive rate of Ki67, a proliferation marker, in TRIM55-overexpressing tumors (Fig. 4C). We assessed the impact of TRIM55 on in vivo angiogenesis and observed that subcutaneous tumors formed by TRIM55-overexpressed Huh7 cells exhibited decreased levels of CD34, a marker indicative of microvascularity, when contrasted with those formed by control cells (Fig. 4C).

To evaluate the effect of TRIM55 on the metastasis of HCC in vivo, the control and TRIM55-overexpressed Huh7 cells were injected into the tail vein of nude mice. After 8 weeks, the metastatic nodules in lung tissues were checked using H&E staining. As expected, TRIM55 overexpression group showed less and smaller metastatic nodules compared to the control group (Fig. 4D). Collectively, these findings strongly indicate that TRIM55 suppresses malignant behaviors of HCC cells in vivo.

### TRIM55 interacts with NF90 and promotes its degradation

**Fig. 5:**
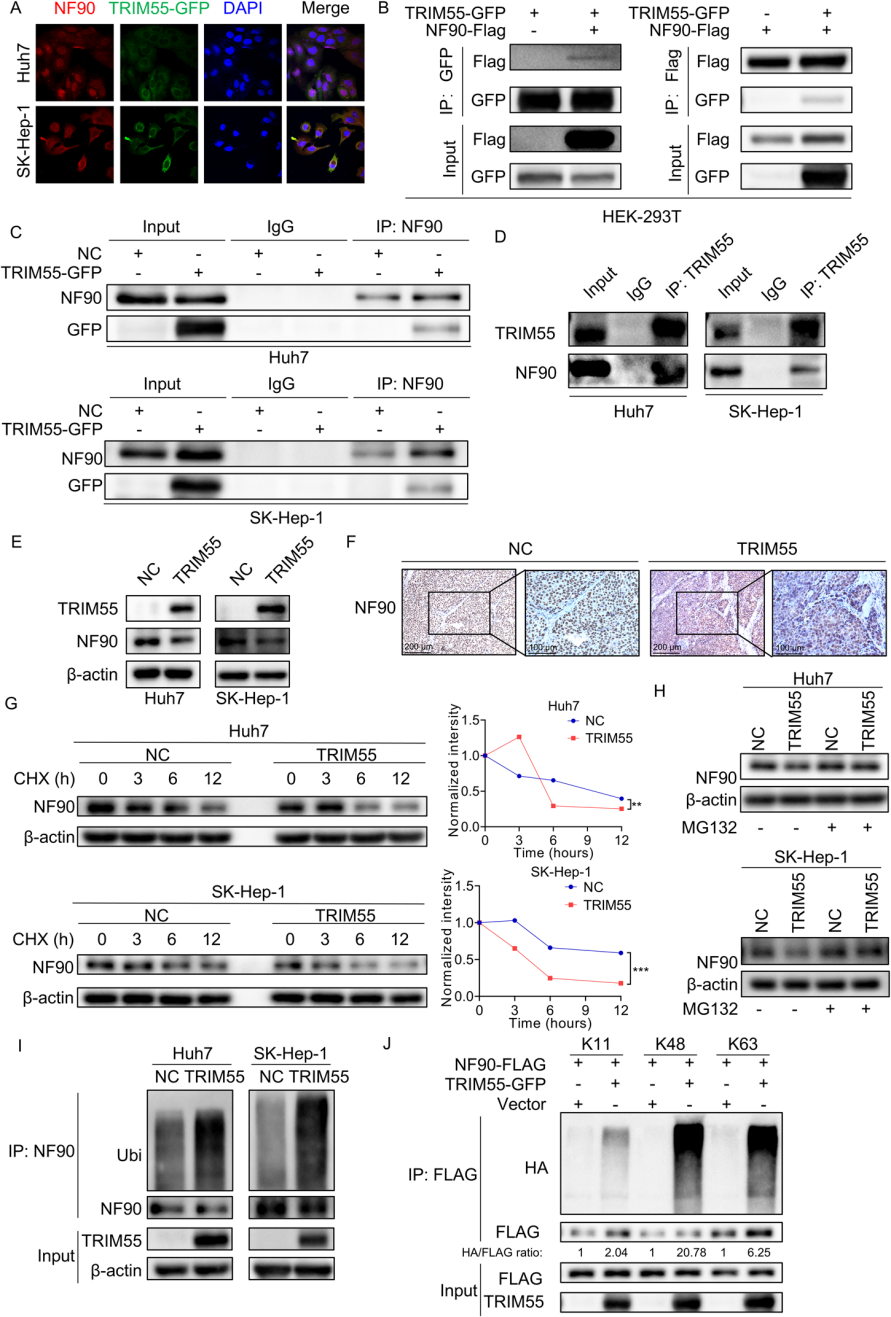
TRIM55 interacts with and ubiquitinates NF90. **A** The cells transfected with GFP-tagged TRIM55 were immunostained for NF90 (red), and DNA (DAPI, blue). Representative fluorescence images of NF90 and TRIM55 localization in cells are shown in the figure. There is space for co-positioning between NF90 and TRIM55. **B** Co-IP assay was performed in HEK-293T cells transfected with TRIM55-GFP and NF90-Flag to explore the interaction between exogenous TRIM55 and NF90. **C** Co-IP experiments were conducted in HCC cells with TRIM55 overexpression to explore the interaction between exogenous TRIM55 and endogenous NF90. **D** Endogenous co-IP experiments indicated that TRIM55 interacted with NF90 in Huh7 and SK-Hep-1 cells. IgG was used as a negative antibody. **E** NF90 expression levels in control and TRIM55 overexpressing HCC cells were determined via western blotting. **F** IHC analysis of the expression of NF90 proteins in control and TRIM55-overexpressed xenografts. **G** Control and TRIM55 overexpressing HCC cells were treated with CHX (200 μg/ml) for time intervals indicated. The cell lysates were subjected to immunoblotting (left panel) and NF90 expression level was quantified (right panel). **H** Control and TRIM55 overexpressing HCC cells were treated with MG132 (10 µM) for 8 h. Then, the NF90 was examined using western blotting. **I** Control and TRIM55 overexpressing HCC cells were treated with MG132 10 µM for 8 h. Cell lysates were then subjected to immunoprecipitation with an anti-NF90 antibody. The resulting immunocomplexes were immunoblotted with anti-NF90 and anti-ubiquitin antibodies. **J** HEK293T cells overexpressing FLAG-NF90, GFP-TRIM55, and HA-tagged different forms of ubiquitin were treated with MG132 (10 μM) for 8 h. The cell lysates were immunoprecipitated by using anti-FLAG M2 affinity gel. The ubiquitination levels of NF90 were detected using anti-HA antibody. *n* = 3, **P < 0.01, ***P < 0.001.

Our previous investigation has unveiled that USP11 forms a binding with NF90, facilitating its deubiquitination process. This, in turn, prevents NF90’s degradation and fosters the progression of HCC [14]. A recent study has discovered that TRIM32, another member of the TRIM family, competes with USP11 to maintain a balance between ubiquitination and deubiquitination of the tumor suppressor gene ARID1A. This competition ultimately promotes or inhibits the metastatic potential of cancer cells, respectively [26]. However, it remains unknown whether the TRIM family can regulate the ubiquitination modification of NF90 in opposition to USP11. Intriguingly, a high-throughput cytosolic yeast analysis from a previous study has identified that both TRIM63 (also known as MuRF1) and TRIM55 may form associations with NF90 [25]. Furthermore, through an analysis conducted using the Kaplan–Meier Plotter online tool, we observed that the expression of TRIM55, but not TRIM63, exhibits a significant correlation with the prognosis of patients with HCC (Supplemental Fig. 1A, B). As a result, our objective is to delve into the potential regulatory relationship between TRIM55 and NF90. As none of the available TRIM55 antibodies conformed to our immunofluorescence (IF) quality criteria, we generated a GFP-tagged TRIM55 construct, which was transfected into HCC cells. Upon the exogenous overexpression of TRIM55-GFP in huh7 and SK-Hep-1 cells, we observed that substantial TRIM55-GFP co-localized with NF90 protein (Fig. 5A). Subsequent exogenous co-immunoprecipitation (co-IP) experiments were conducted in both HEK-293T and HCC cells (Fig. 5B, C), revealing a significant association between TRIM55 and NF90. This interaction was further confirmed through endogenous co-IP assays in HCC cells (Fig. 5D, Supplemental Fig. 2A).

To elucidate the regulatory role of TRIM55 in NF90, we examined the mRNA and protein levels of NF90 in HCC cells overexpressing TRIM55. Our findings revealed that TRIM55 did not influence the mRNA expression of NF90 (Supplemental Fig. 1C), but notably reduced its protein levels (Fig. 5E). This observation was further supported by the attenuated NF90 IHC staining in TRIM55-overexpressed xenografts of nude mice (Fig. 5F). These results suggest that TRIM55 may be involved in the degradation of NF90. To assess the impact of TRIM55 overexpression on the stability of endogenous NF90 protein, we conducted a cycloheximide (CHX) pulse-chase assay. HCC cells, with or without TRIM55 overexpression, were treated with CHX to inhibit protein synthesis for a specified duration. Subsequently, TRIM55 protein levels were evaluated through western blotting. Notably, we observed that TRIM55 overexpression expedited the degradation of NF90 protein in both Huh7 and SK-Hep-1 cells (Fig. 5G). The administration of the proteasome inhibitor MG132 abrogated the effect of TRIM55 on NF90 protein levels (Fig. 5H). Moreover, in vivo ubiquitination experiments confirmed that TRIM55 overexpression significantly augmented the ubiquitination level of NF90 (Fig. 5I). As anticipated, knockdown of TRIM55 upregulated NF90 expression, attenuated its degradation and ubiquitination modification in PLC/PRF/5 cells (Supplemental Fig. 2B–D).

Furthermore, the specific types of polyubiquitination modifications of NF90 proteins affected by TRIM55-mediated ubiquitination was investigated. HEK-293T cells were transfected with TRIM55-GFP, NF90-FLAG, and each of the different ubiquitins (K11-, K48-, or K63-only ubiquitin-HA) individually. Immunoprecipitation of FLAG-NF90 proteins from the lysates was followed by western blot analysis using an anti-HA antibody. Our findings demonstrated a substantial increase in K11-, K48-, and K63-linked ubiquitination on NF90 due to TRIM55 overexpression (Fig. 5J). Among these three types, K48-linked ubiquitination on NF90 exhibited the most significant impact. Collectively, these data suggest that TRIM55 regulates the stability of NF90 through the ubiquitin-proteasome pathway.

### TRIM55 inhibits HCC progression in part through the modulation of NF90

**Fig. 6:**
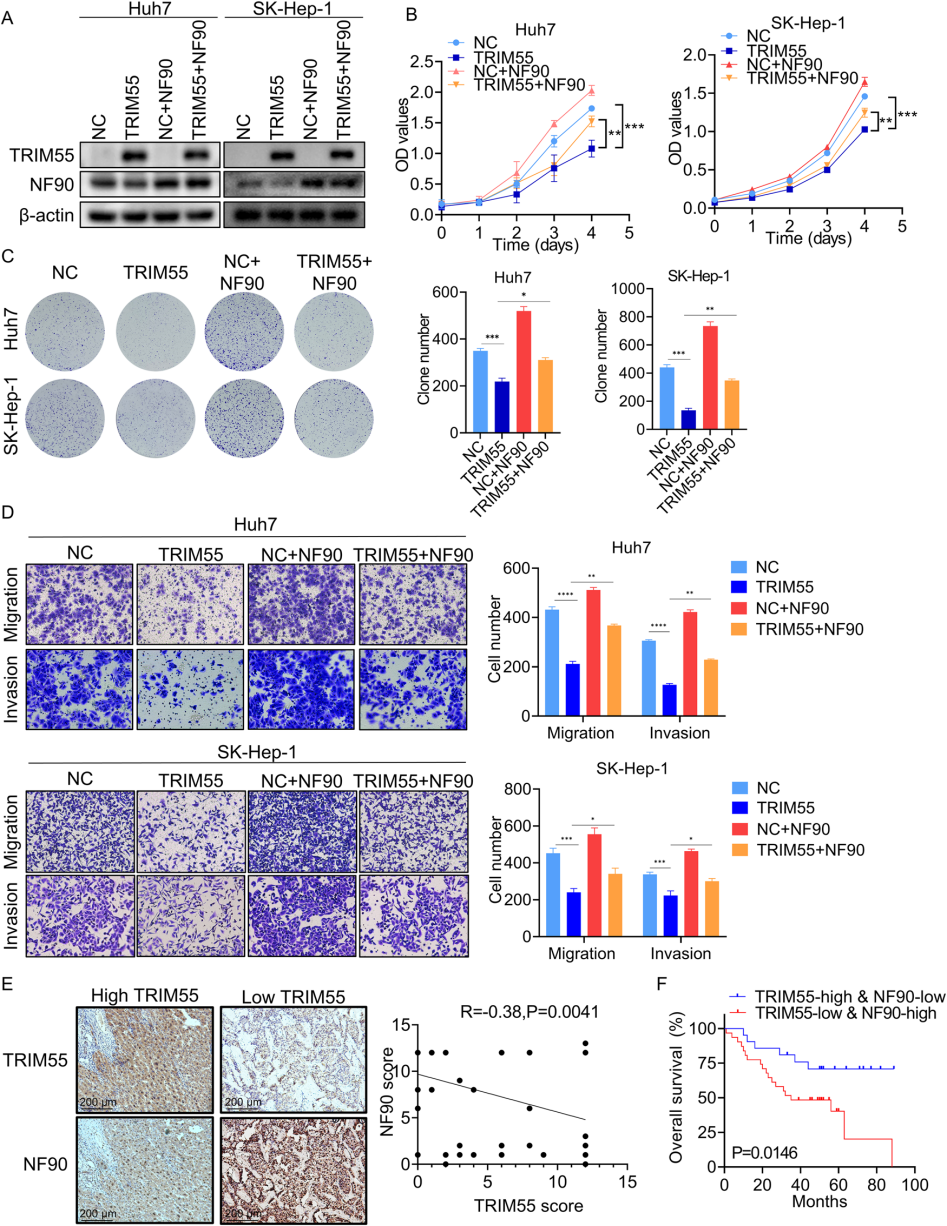
TRIM55 inhibits HCC cells proliferation and metastasis partially via NF90. **A** The efficacy of TRIM55 overexpression and NF90 reintroduction was validated via immunoblotting analysis. **B** NF90 partially abolished the TRIM55-induced inhibition of proliferation in HCC cells as confirmed by CCK-8 assays. **C** NF90 partially abolished the TRIM55-induced inhibition of clone formation ability in HCC cells as confirmed by CCK-8 assays. **D** NF90 partly impaired the TRIM55-induced inhibition of migration and invasion in HCC cells as confirmed by transwell assays. **E** Representative images of IHC staining of NF90 in HCC tissues with different levels of TRIM55. Scatter diagram exhibited a negative correlation of TRIM55 and NF90 expression in HCC tissues. **F** The patients were categorized into the TRIM55-low & NF90-high and TRIM55-high & NF90-low groups based on IHC staining. Survival analysis of HCC patients by log-rank tests was performed. *n* = 3, *P < 0.05, **P < 0.01, ***P < 0.001, ****P < 0.0001.

To investigate the potential influence of TRIM55 on HCC progression in a manner dependent on NF90, rescue experiments were performed by reintroducing NF90 into HCC cells that were overexpressing TRIM55 (Fig. 6A). Through CCK-8, colony formation, and transwell assays, it was demonstrated that the upregulation of NF90 expression partially counteracted the inhibitory effect of TRIM55 on HCC cell proliferation, migration, and invasion (Fig. 6B–D). To further confirm the pathological association between TRIM55 and NF90 expression, IHC staining of NF90 and TRIM55 was performed on the same set of HCC tissues. The downregulation of TRIM55 expression in HCC tissues coincided with the upregulation of NF90, exhibiting a significant negative correlation between their expression levels (Fig. 6E). Furthermore, the Kaplan–Meier method was employed to analyze the impact of combined TRIM55 and NF90 expression on the clinical outcomes of HCC patients, revealing that the TRIM55-low & NF90-high group exhibited a worse prognosis compared to the TRIM55-high & NF90-low group (Fig. 6F). Collectively, our findings underscore the significance of NF90 in the tumor-suppressive role of TRIM55 in HCC.

### TRIM55 promotes the degradation of HIF1α mRNA

**Fig. 7:**
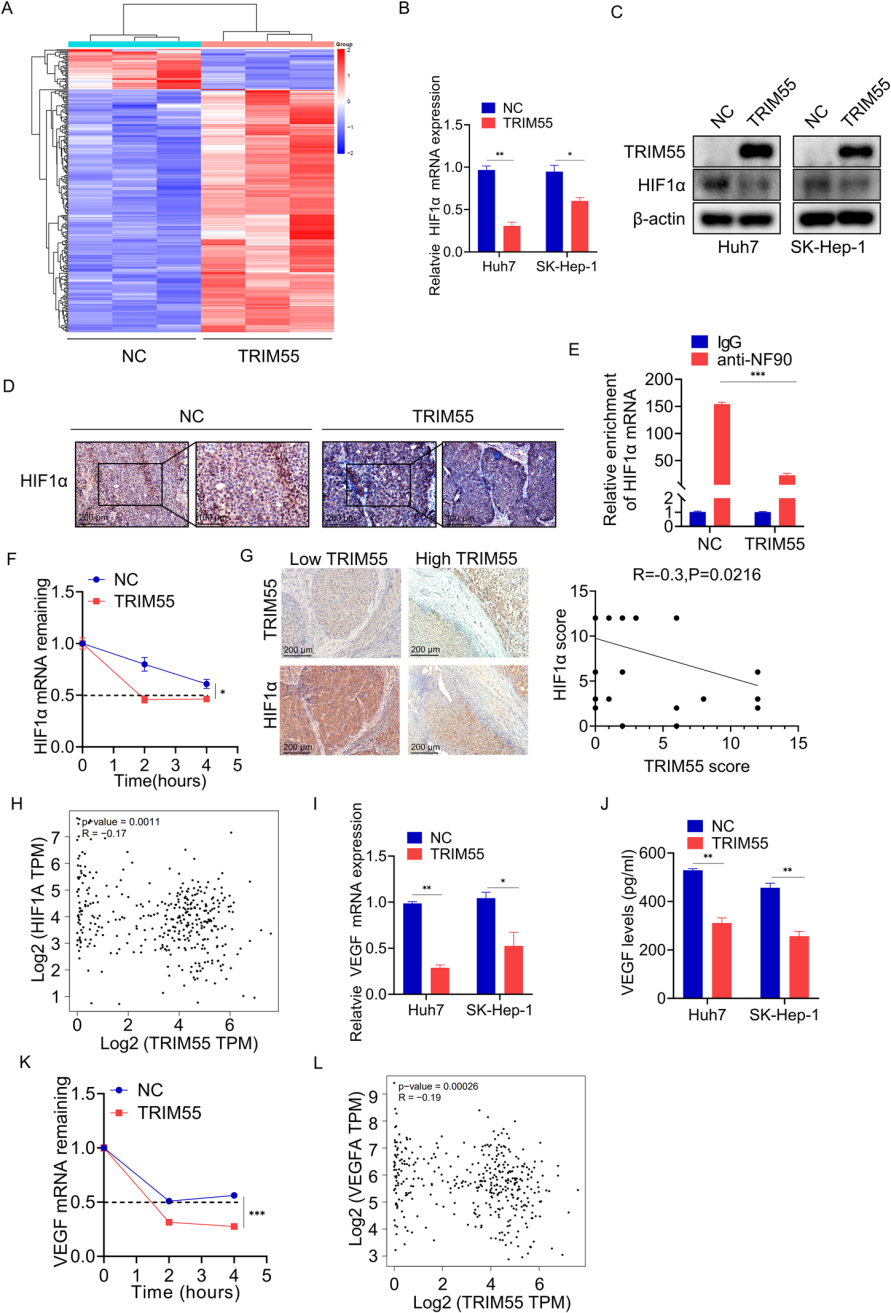
TRIM55 regulates the HIF1α/VEGF pathway. **A** Heatmap of differentially expressed genes in control and TRIM55 overexpressing Huh7 cells. **B** qRT-PCR analysis of HIF1-α mRNA expression in Huh7 and SK-Hep-1 cells transfected empty vector and TRIM55. **C** Westen blot analysis of HIF1-α protein level in Huh7 and SK-Hep-1 cells transfected empty vector and TRIM55. **D** Expression of HIF1α in xenografts formed by control and TRIM55-overexpressed Huh7 cells was detected by IHC. **E** RIP assay was used to detect the binding of NF90 to HIF1α in control and TRIM55-overexpressed Huh7 cells. **F** The Huh7 cells were treated with actinomycin D (ActD, 5 mg/mL) for 0, 2, 4 h. The HIF1α mRNA level was measured using qRT-PCR and normalized to 18S rRNA. **G** Representative images of IHC staining of HIF1α in HCC tissues with different expression levels of TRIM55. Scatter diagram exhibited a negative correlation of TRIM55 and HIF1α in HCC tissues by IHC. **H** TCGA database were used to analyze the relationship between the expression of TRIM55 and HIF1α mRNA. **I** qRT-PCR analysis of VEGF mRNA expression in Huh7 and SK-Hep-1 cells transfected empty vector and TRIM55. **J** ELISA assay was performed to test the VEGF protein expression in Huh7 and SK-Hep-1 cells transfected empty vector and TRIM55. **K** The Huh7 cells were treated with actinomycin D (ActD, 5 mg/mL) for 0, 2, 4 h. The VEGF mRNA level was measured using qRT-PCR and normalized to 18S rRNA. **L** Correlation analysis between TRIM55 and VEGF in HCC samples from TCGA database. *n* = 3, *P < 0.05, **P < 0.01, ***P < 0.001.

To explore the downstream signaling regulated by TRIM55 in greater depth, we conducted RNA sequencing in control and TRIM55-overexpressed Huh7 cells (Fig. 7A). We discovered that TRIM55 upregulated 236 genes and downregulated 39 genes (|log2(foldchange)| > 1, padj < 0.05). Gene Ontology (GO) and Kyoto Encyclopedia of Genes and Genomes (KEGG) analysis revealed that these differentially expressed genes were significantly enriched in cancer-regulated pathways, such as biological adhesion, cell adhesion, cell migration, tight junction, and cell surface receptor signaling pathway (Supplemental Fig. 3A, B). Hypoxia-inducible factor 1 alpha (HIF1α), a transcription factor that regulates the cellular response to hypoxia and is upregulated in HCC, plays a crucial role in growth, metastasis, tumor angiogenesis, and therapy resistance [27]. Previous studies have shown that HIF1α is a genuine downstream effector of NF90, as NF90 associates with its mRNA to prevent degradation [18, 28]. Consistent with this, analysis of the TCGA database and our clinical cohort revealed a positive correlation between NF90 and HIF1α (Supplemental Fig. 4A, B). Furthermore, our RNA sequencing results demonstrated that TRIM55 overexpression significantly affected HIF1α. Therefore, we investigated whether HIF1α functions as a downstream regulator of TRIM55 via NF90. To validate this, we measured the mRNA and protein levels of HIF1α upon TRIM55 overexpression using qRT-PCR and western blot experiments. The results showed that HCC cells overexpressing TRIM55 exhibited lower HIF1α expression compared to the control group (Fig. 7B, C). IHC staining of HIF1α in xenografts of nude mice further supported this finding (Fig. 7D). Moreover, we conducted a RIP assay followed by qRT-PCR to examine the potential impact of TRIM55 on the interaction between NF90 and HIF1α mRNA. Notably, upon overexpression of TRIM55, a noticeable decrease in the affinity between NF90 and HIF1α mRNA was observed compared to the control group (Fig. 7E). To assess the influence of TRIM55 on the stability of HIF1α mRNA, we treated Huh7 cells with Actinomycin D, an inhibitor of new RNA synthesis, in both the control and TRIM55-overexpressed groups. Subsequently, we monitored the decline in HIF1α mRNA expression over a duration of 2 and 4 h. Remarkably, the induction of TRIM55 significantly accelerated the degradation of HIF1α mRNA (Fig. 7F). Additionally, we analyzed the TCGA database and performed IHC on our clinical samples, revealing a negative correlation between TRIM55 and HIF1α (Fig. 7G, H), thus providing support for HIF1α being a downstream gene of TRIM55.

VEGF serves as a fundamental constituent of angiogenesis and represents a critical target of HIF1α, whose mRNA stability is upheld by HIF1α. Given our discovery of TRIM55’s functional role in angiogenesis regulation, we sought to investigate its potential to modulate VEGF. The outcomes of qRT-PCR and enzyme-linked immunosorbent assay (ELISA) unequivocally revealed that TRIM55 possessed the ability to downregulate both VEGF mRNA and protein expression (Fig. 6I, J). Notably, cells overexpressing TRIM55 exhibited a noticeably shorter half-life of VEGF mRNA compared to control cells (Fig. 6K). Furthermore, analysis of the TCGA database confirmed a negative correlation between TRIM55 expression and VEGF levels in tumor tissues (Fig. 6L), thereby providing additional support for TRIM55’s regulation of VEGF. Collectively, these findings conclusively demonstrate that TRIM55 represses the HIF1α/VEGF signaling pathway in HCC cells.

### TRIM55 suppresses the TGF-β/Smad signaling

**Fig. 8:**
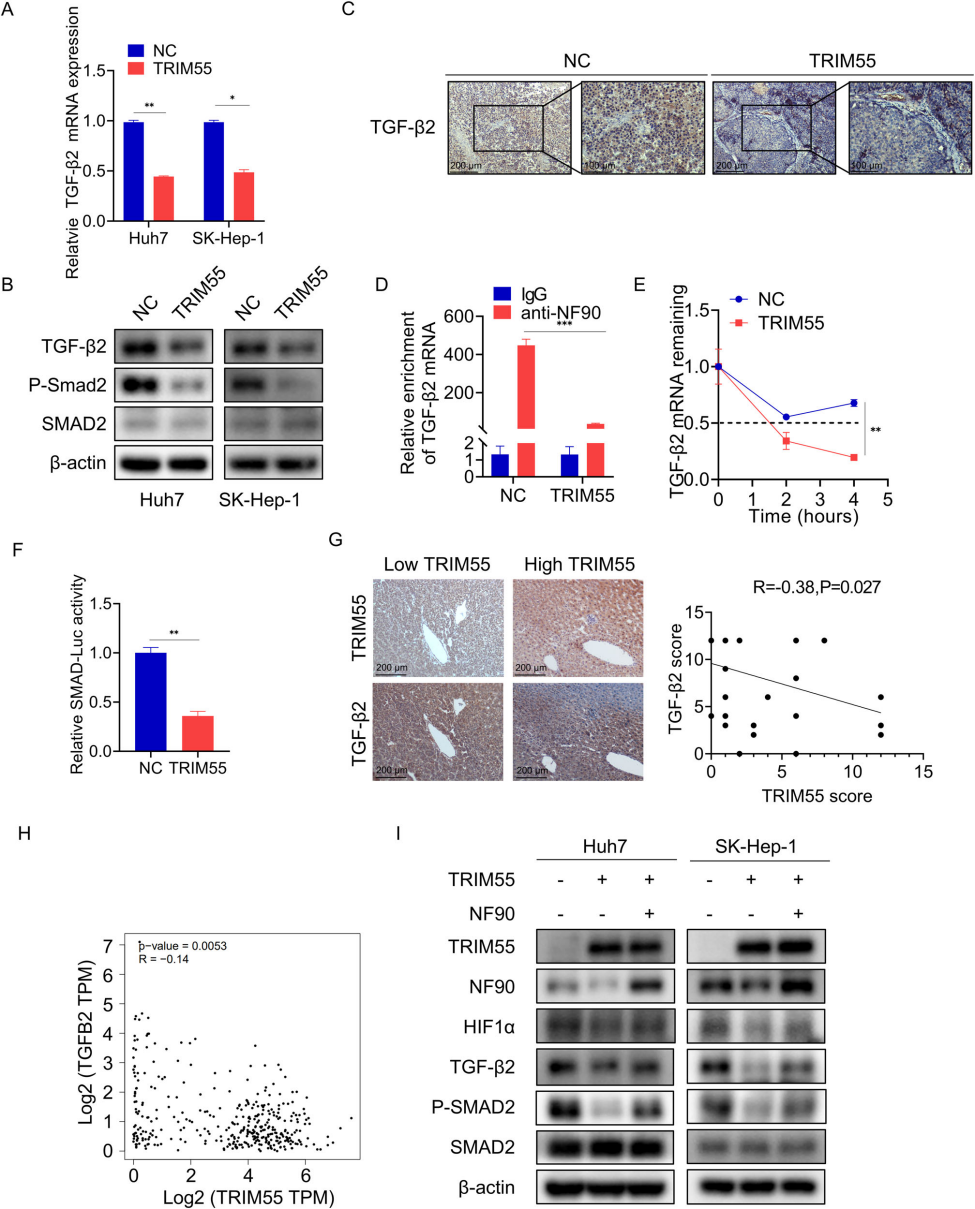
TRIM55 regulates the TGFβ/Smad pathway. **A** qRT-PCR analysis of TGF-β2 mRNA expression in Huh7 and SK-Hep-1 cells transfected empty vector and TRIM55. **B** Westen blot analysis of TGF-β2, Smad2, and P-smad2 protein levels in Huh7 and SK-Hep-1 cells transfected empty vector and TRIM55. **C** Expression of TGF-β2 in xenografts formed by control and TRIM55-overexpressed Huh7 cells was detected by IHC. **D** RIP assay was used to detect the binding of NF90 to TGF-β2 mRNA in control and TRIM55-overexpressed Huh7 cells. **E** The Huh7 cells were treated with ActD (5 mg/mL) for 0, 2, 4 h. The TGF-β2 mRNA level was measured using qRT-PCR and normalized to 18S rRNA. **F** HEK-293T cells were co-transfected with TRIM55 and SMAD-Luc reporter. 24 h later, the luciferase activity was measured. **G** Representative images of IHC staining of TGF-β2 in HCC tissues with different expression levels of TRIM55. Scatter diagram exhibited a negative correlation of TRIM55 and HIF1α in HCC tissues by IHC. **H** Correlation analysis between TRIM55 and TGF-β2 in HCC samples from TCGA database. **I** The NF90 was transfected into Huh7 and SK-Hep-1 cells with TRIM55 knockdown. Then, western blot detecting indicated proteins was performed. *n* = 3, *P < 0.05, **P < 0.01, ***P < 0.001.

We then gained insight into the involvement of other cancer-related pathways modulated by TRIM55-NF90. The TGF-β signaling pathway serves as a pro-tumorigenic signal, triggering the manifestation of aggressive tumor characteristics, such as epithelial-mesenchymal transition (EMT), remodeling of the tumor microenvironment, and immune evasion by cancer cells [29–31]. Among the differentially expressed genes regulated by TRIM55, as revealed by our RNA-sequencing analysis, TGF-β2 appeared to be significantly affected by TRIM55. The analysis of the TCGA database and our clinical cohort unveiled a positive correlation between NF90 and TGF-β2 (Supplemental Fig. 4C, D), suggesting that TGF-β2 might represent a potential target of TRIM55-NF90. To validate this hypothesis, we assessed the expression of TGF-β2 in cells and xenografts with or without TRIM55 overexpression and observed a reduction in TGF-β2 expression following TRIM55 overexpression (Fig. 8A–C). Furthermore, NF90 was found to bind to TGF-β2 mRNA, which could be significantly inhibited by TRIM55 overexpression (Fig. 8D). Consistently, the upregulation of TRIM55 expedited the degradation of TGF-β2 mRNA compared to the control group (Fig. 8E). Phosphorylated Smad2 protein (P-Smad2) elevation is considered an indicator of TGFβ signaling activation. To investigate whether TRIM55 overexpression inactivated the TGFβ/Smad pathway, we examined Smad2 and P-Smad2 levels via western blot analysis. A decrease in P-Smad2 expression was observed in HCC cells with TRIM55 overexpression (Fig. 8B). Additionally, TRIM55 exerted a significant inhibitory effect on the activity of the Smad-Luc reporter (Fig. 8F). Subsequently, we investigated the pathological association between TRIM55 and TGF-β2. We evaluated the expression of TRIM55 and TGF-β2 in the same cohort of HCC tissues, revealing an inverse correlation between them (Fig. 8G). Furthermore, analysis of the TCGA database further validated the negative correlation between TRIM55 and TGF-β2 expression (Fig. 8H). These findings suggest that TRIM55 induces deactivation of the TGFβ/Smad signaling pathway.

Then, we examined the significance of NF90 in TRIM55-regulated HIF1α and TGF-β2. As demonstrated by the results of western blot, restoration of NF90 expression partially rescued the TRIM55-mediated downregulation of HIF1α, TGF-β2 and P-Smad2 expression (Fig. 8I), suggesting that TRIM55 regulated HIF1α and TGF-β2 partially via NF90 in HCC cells.

### TRIM55 inhibits HCC progression partially through HIF1α and TGF-β2

**Fig. 9:**
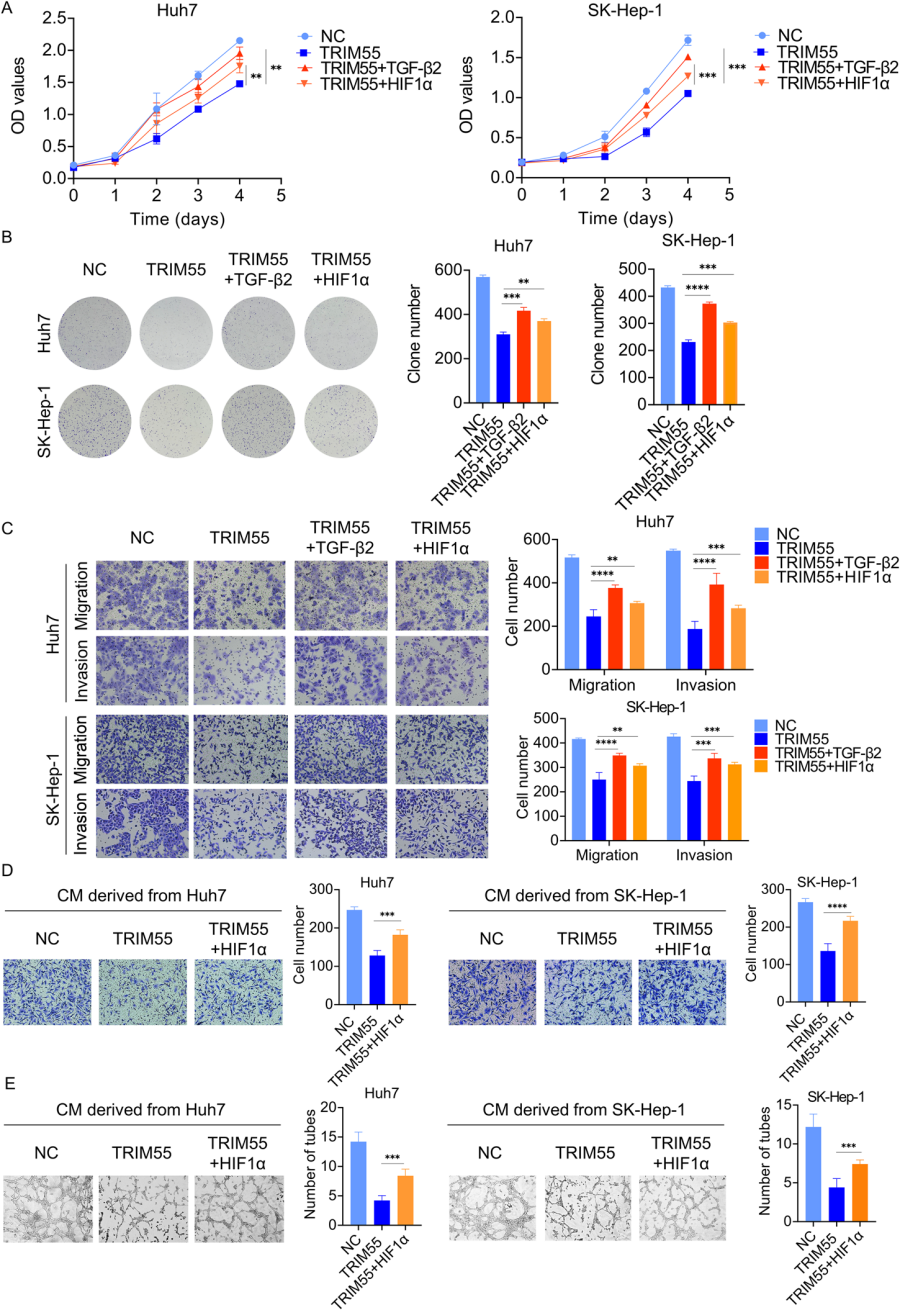
TRIM55 inhibits HCC progression partially through HIF1α and TGF-β2. **A** The CCK-8 assay detecting the proliferation of indicated Huh7 and SK-Hep-1 cells. **B** The colony formation assay detecting the proliferation of indicated Huh7 and SK-Hep-1 cells. **C** The transwell assay detecting the migration and invasion of indicated Huh7 and SK-Hep-1 cells. **D** Transwell assay testing the HUVECs migration affected by the CM derived from indicated Huh7 and SK-Hep-1 cells. **E** HUVECs tube formation affected by the CM derived from indicated Huh7 and SK-Hep-1 cells. *n* = 3, **P < 0.01, ***P < 0.001, ****P < 0.0001.

Finally, we investigated whether HIF1α and TGF-β2 is involved in the suppressive effect of TRIM55. TRIM55-ovexpressd Huh7 and SK-Hep-1 cells were transfected with HIF1α or treated with TGF-β2 (Supplemental Figs. 4D, 5C). As evidenced by the results of CCK-8, colony formation and transwell experiments, both HIF1α overexpression and the treatment of TGF-β2 was capable to partially rescue the TRIM55-mediated suppression of cell proliferative, migratory, and invasive ability (Fig. 9A–C). Since HIF1α plays a critical role in tumor angiogenesis, we also examined whether the anti-angiogenesis role of TRIM55 is dependent on HIF1α. As expected, the migration and tube formation of HUVECs was attenuated by the CM derived from TRIM55-overexpressed HCC cells, which was partly abolished by HIF1α overexpression (Fig. 9D, E). Taken together, our results indicate that the enforced expression of HIF1α and TGF-β2 can counterbalance the tumor-suppressive impact of TRIM55 in HCC.

## Discussion

In our investigation, we discovered that TRIM55 exhibited reduced expression in HCC tissues. The diminished levels of TRIM55 were significantly associated with larger tumor size and elevated serum AFP levels. Moreover, this downregulation served as a reliable indicator of unfavorable overall and tumor-free survival in HCC patients. Our analysis of the TCGA database further revealed that the decreased expression of TRIM55 in HCC patients was indicative of a tendency towards a poor prognosis, aligning with our own findings. A previous study also confirmed the decline in TRIM55 expression in HCC and its correlation with an unfavorable clinical outcome [32]. TRIM55 exerted inhibitory effects on the proliferation, migration, and invasion of HCC cells in vitro, and effectively suppressed tumor growth in vivo. Furthermore, we unveiled a novel role of TRIM55 in impeding tumor angiogenesis. These findings unequivocally establish TRIM55 as a potent tumor suppressor and a promising prognostic biomarker for patients with HCC.

Several protein substrates of TRIM55 have already been elucidated. Snail1, the pivotal transcriptional regulator of EMT process, has been identified as a substrate of TRIM55 in lung adenocarcinoma cells. TRIM55 facilitates the ubiquitination of Snail1, thereby expediting its degradation via the ubiquitin-proteasome pathway [10]. C-Myc, a prominent oncogenic transcription factor, governs target genes implicated in diverse pathways encompassing cellular proliferation, metabolism, and immune evasion. It assumes a pivotal role in the initiation and progression of tumors across multiple cancer types [33]. In colorectal cancer, TRIM55 targets c-Myc and downregulates its protein level via ubiquitination, thereby suppressing tumor growth [11]. However, the specific protein substrate of TRIM55 in HCC remains unidentified. In this study, NF90 emerged as a downstream target of TRIM55. Our findings unveiled the colocalization of TRIM55 with NF90, accompanied by their direct interaction. Notably, TRIM55 exhibited no influence on the mRNA expression of NF90, yet it exerted a downregulating effect on its protein abundance. This downregulation was effectively counteracted by the administration of the proteasome inhibitor MG132. Furthermore, TRIM55 was found to enhance the ubiquitination level of NF90 protein. Collectively, these observations strongly suggest that TRIM55 promotes the degradation of NF90 via the ubiquitin-proteasome pathway. Ubiquitin molecules are linked through different lysine residues to form various types of polyubiquitination. Polyubiquitination at lysine 11 (K11) and K48 primarily function in protein degradation and regulation of protein stability. On the other hand, polyubiquitination at K63 mainly regulates signal transduction, DNA repair, and protein activity. Additionally, it can also impact protein stability [34–36]. Here, we have showcased a remarkable augmentation in the ubiquitination of NF90, specifically in the forms of K11, K48, and K63 linkages, as a result of TRIM55 overexpression. Notably, K48-linked ubiquitination on NF90 exhibited the most profound influence. Previous research has indicated that TRIM47 interacts with the zinc finger motif of NF90, leading to proteasome-mediated degradation through K48-linked ubiquitination at Lys297. Whether TRIM55-mediated diverse forms of polyubiquitination also regulate this particular site of NF90 necessitates further investigation. Our previous study has revealed a significant association between the increased expression of deubiquitinase USP11 and unfavorable prognosis in patients with HCC, as well as its role in promoting the progression of HCC. The regulation of NF90 deubiquitination by USP11 is crucial for the enhanced proliferation and metastasis of HCC [24]. In this study, we have demonstrated that TRIM55 partially impedes HCC progression by inhibiting NF90. These aforementioned findings underscore the balance between the E3 ligase TRIM55 and deubiquitinase USP11 in maintaining the stability of NF90, thereby controlling the oncogenic or tumor-suppressive state of HCC cells. Specifically, TRIM55 facilitates the degradation of NF90 to suppress HCC progression, while USP11 stabilizes NF90 to exert its oncogenic functions.

Hypoxia, a defining characteristic of the solid tumor microenvironment, arises from aberrant tumor microvessels, obstructed microcirculation, and compromised diffusion, resulting in inadequate or absent oxygen supply within the tumor microenvironment [37]. The activation of the hypoxia-inducible factor (HIF), a transcription factor, empowers cancer cells to adapt to the oxygen-deprived environment by transactivating downstream target genes. Within the human genome, three distinct subtypes of HIF are encoded: HIF1α, HIF2α, and HIF3α. The expression of hypoxia-induced genes predominantly hinges upon the stability of the α subunit of HIF1α, an oxygen-labile component whose transcriptional efficacy is governed by cellular oxygen tension [38–40]. The expression of HIF1α is markedly elevated in HCC tissues. Enhanced levels of HIF-1α have been recognized as a prognostic marker, associated with an unfavorable prognosis. Functionally, HIF-1α promotes HCC proliferation, stemness, invasion, EMT, metastasis, angiogenesis, and immune escape. The upregulation of HIF-1α can be attributed to aberrant transcriptional, post-transcriptional, or translational regulation in HCC [41–44]. NF90 has been identified as a regulator of HIF1α, as it forms associations with and enhances the stability of HIF1α mRNA [18, 28]. In this study, we presented evidence that TRIM55 suppressed the expression of HIF1α and its downstream target gene VEGF. TRIM55 attenuated the interaction between NF90 and HIF1α mRNA, thereby expediting the degradation of HIF1α mRNA. Additionally, the inverse correlation observed between TRIM55 and HIF1α expression in HCC tissues provided further support for the notion that HIF1α acts as a downstream mediator of TRIM55.

The regulatory cytokine TGF-β and its signaling effectors govern a wide range of molecular and cellular responses that are precisely regulated in space and time. However, paradoxically, they play dual and opposing roles in the progression of HCC. In the early stages of tumorigenesis, TGF-β signaling exerts profound tumor-suppressive effects by inducing cell cycle arrest, cellular senescence, autophagy, and apoptosis. Nevertheless, as the tumor advances towards malignancy, TGF-β undergoes a functional switch, activating a pro-tumorigenic signal that triggers aggressive tumor characteristics, including EMT, remodeling of the tumor microenvironment, and immune evasion by cancer cells [29–31]. By employing transcriptome sequencing and subsequent verification, we ascertained that TRIM55 effectively suppressed the expression of TGF-β2 and the activation of P-Smad2, a key indicator of TGFβ signaling. Additionally, TRIM55 attenuated the association between NF90 and TGF-β2 mRNA, thereby facilitating its degradation. Our analysis of clinical specimens and the TCGA database revealed an inverse relationship between TRIM55 and TGF-β2 expression, indicating that TRIM55 also governs the TGFβ/Smad signaling pathway. In a recent study, it was discovered that USP11 has the ability to enhance the stability of TGFβ receptor type 2 (TGFBR2) and initiate downstream TGFβ signaling in cancer cells [45]. This finding leads us to hypothesize that, in addition to NF90, both USP11 and TRIM55 may exert distinct effects on the activation and suppression of the TGFβ/Smad signaling, which potentially play pivotal roles in the progression of HCC.

## Conclusion

In summary, our study revealed that decreased TRIM55 expression in HCC exhibits a significant association with unfavorable prognosis. TRIM55 assumes a paramount function in suppressing the progression of HCC partially through the degradation of NF90 and the subsequent inactivation of its downstream pathway, encompassing HIF1α/VEGF and TGFβ/Smad signaling.

## Materials and methods

### Tissue samples collection

HCC and corresponding adjacent nontumor tissues, as well as follow-up data, were obtained from the Department of Hepatobiliary Surgery, Zhongshan Hospital of Xiamen University. All clinical tissue samples were collected under informed consent of the patients, and the study protocols were approved by the Ethics Committee of the Zhongshan Hospital of Xiamen University.

### Cell culture

HEK-293T, HUVEC, PLC/PRF/5, Huh7, and SK-Hep-1 cell lines were purchased from Cellcook Biotechnology Company (Guangzhou, China), and cultured in high glucose Dulbecco’s modified Eagle medium (DMEM; Gibco) supplemented with 10% fetal bovine serum (FBS; Vivacell) and 1% penicillin/streptomycin (Gibco), and in a humidified incubator under 5% CO_2_ at 37 °C. Cells were tested for mycoplasma contamination and the authentication of these cell lines was performed via comparisons with the STR database.

### Plasmid construction and lentivirus preparation

For TRIM55 overexpression, full-length TRIM55 was cloned into the lentiviral vector pLV-EGFP-C (Inovogen Tech. Co.). For TRIM55 knockdown, two different shRNAs targeting TRIM55 were inserted into the lentiviral vector pLV-shRNA-puro (Inovogen Tech. Co.). Lentivirus-expressing vectors and control plasmids were co-transfected with psPAX2 and pMD.2G into HEK-293T cells. After 24 h, lentiviral particles were collected and were used to infect HCC cells with 5 μg/mL polybrene (Solarbio, Beijing, China). Stable cells were selected using 2 μg/mL puromycin (Solarbio, Beijing, China) for 1 week.

### Immunohistochemical (IHC) staining

Tissue was fixed with 10% formalin and embedded in paraffin and cut into 3-µm sections for IHC staining. The sections were stained with anti-TRIM55 (1:200, Abcam), anti-NF90 (1:2000, Abclonal), anti-HIF1α (1:400, Proteintech), anti-CD34 (1:3000, Abcarm), anti-Ki67 (Maixin) and anti-TGF-β2 (1:1000, Proteintech) antibodies at 4 °C overnight, incubated with a secondary antibody for 30 min at room temperature, developed with diaminobenzidine, and stained with hematoxylin. The IHC reagents were purchased from Maixin Biotechnology (Fuzhou, China). The tissue sections were scored according to the degree of staining (0–3 points are negative coloring, light yellow, light brown, and dark brown) and the positive scale (1–4 points are 0–25%, 26–50%, 51–75%, and 76–100%). Finally, the scores were calculated as the product of degree of staining and the positive scale. The above analysis methods were reviewed and performed double-blind analysis by two independent pathologists. The score ranging from 0 to 6 was considered as a low-expression group, whereas the score ranging from 7 to 12 was considered as a high-expression group.

### Western blot

Proteins were extracted using RIPA lysis buffer (Beyotime) containing a phosphatase inhibitor (LABLEAD) and a protease inhibitor (LABLEAD). The proteins were separated via 10% SDS-PAGE and transferred onto polyvinylidene fluoride (PVDF) membranes (Millipore). The PVDF membranes were blocked with BSA for at least 1 h at room temperature and then incubated with primary antibodies overnight at 4 °C, followed by incubation with corresponding secondary antibody and visualization with ECL Western Blotting Substrate (Millipore). Western blot analysis was performed using the following antibodies: anti-β-actin (Cell Signaling), anti-TRIM55 (Abcam), anti-NF90 (Abclonal), anti-HIF1α (Abclonal), SMAD2 (Abclonal), anti-phospho-SMAD2 (Abclonal) and anti-TGF-β2 (Proteintech). The results were observed using an ECL detection system (Millipore). The original uncropped images of western blot were shown in Supplemental Fig. 4.

### Protein half-life assays

Cells in the exponential growth period were treated with cycloheximide (CHX) (200 μg/ml) for different times to block protein synthesis. The cells were harvested and subjected to immunoblot analysis using antibodies against NF90 and β-actin to observe changes in protein levels.

### Cell proliferation analysis

To explore the effects of TRIM55 on cell proliferation, 2 × 10^3^ cells were seeded in 96-well plates with 100 µl of culture medium. At indicated time, 10 µl cell counting kit-8 (CCK-8; Dojindo, Beijing, China) reagent was added into culture medium. After incubation at 37 °C for 1 h, the absorbance was measured at 450 nm using a microplate spectrophotometer (Tecan).

### Plate clone formation assay

A total of 2 × 10^3^ cells were seeded in 6-well plates. After 7–14 days, visible clones were formed. Cells were fixed with 4% paraformaldehyde for 30 min, and dyed with 1.0% crystal violet for 30 min.

### RNA extraction and quantitative real-time PCR (qRT-PCR)

Trizol reagent (Invitrogen, Carlsbad, CA, USA) was used to extract total RNA from cells according to the standard protocol. cDNA was synthesized using PrimeScript^TM^ RT reagent Kit (Vazyme, China). The resulting cDNA was used as template to perform qRT-PCR analysis with Taq Pro Universal SYBR qPCR Mater Mix (Vazyme). The primers used for qRT-PCR were listed as follows: GAPDH, forward: 5′-CTTTGGTATCGTGGAAGGACTC-3′, reverse: 5′-AGTAGAGGCAGGGATGATGT-3′; 18sRNA, forward: 5′-ACACGGACAGGATTGACAGA-3′, reverse: 5′-GGACATCTAAGGGCATCACA-3′; NF90, forward: 5′-AACCATGGAGGCTACATGAAT-3′, reverse: 5′-CGCTCTAGGAAGACCCAAAATC-3′; HIF1α, forward: 5′-CCAACCTCAGTGTGGGTATAAG-3′, reverse: 5′-TTTGATGGGTGAGGAATGGG-3′; VEGF, forward: 5′-CGCAAGAAATCCCGGTATAA-3′, reverse: 5′-AAATGCTTTCTCCGCTCTGA-3′; TGF-β2, forward: 5′-AATCCCTGTGCCGTCTT-3′, reverse: 5′-CCCAAACAACCAACCC-3′.

### Migration and invasion assays

8-µm pore size transwell plates (BD Biosciences) with or without Matrigel were placed into 24-well plates containing 500 µl of medium with 10% FBS. Cells were seeded at a density of 5 × 10^4^ cells were seeded into the upper chamber with 200 µl of serum-free medium. After culture for 24–48 h, the cells in the upper chamber were removed with a cotton swab. The cells across the membrane were fixed with 4% paraformaldehyde for 30 min, stained with crystal violet and finally counted under a light microscope.

### RNA sequencing and gene expression analysis

Total RNA was extracted from cells using TRIzol® reagent (Invitrogen). The A260/A280 absorbance ratio of the RNA samples was measured using a Nanodrop ND-2000 (Thermo Scientific, USA), and RNAs that passed the quality control were used for library construction. The RNA sequencing and data analysis was finished by Shanghai Zhongke NewLife Biotechnology. DESeq2 was used to analyze differentially expressed genes, with default thresholds of |log2FC| > 1 and Padj < 0.05. The resulting differential genes underwent GO and KEGG enrichment analysis. The clusterProfiler R package was used to conduct GO and KEGG pathway enrichment analyses. Significant enrichment of a GO or KEGG function was considered to exist when *P* < 0.05.

### Co-immunoprecipitation(co-IP) assay

Protein was extracted utilizing the method described above. Cell lysates, along with 20 µl of magnetic beads and 2 µg of the specified antibody, were incubated overnight at 4 °C. The mixture was placed upon a magnet, leading to the removal of solid material from the supernatant, which was then subjected to three rounds of washing with a washing buffer. Loading buffer was introduced into the tube and heated for a duration of 10 min at 100 °C. Following this, the immunoreactive complex was collected using a magnet and subjected to SDS-PAGE and immunoblotting analysis.

### Ubiquitination assay

For ubiquitination assays, cells were treated with 10 µM MG132 (MCE) for 8 h to block the proteolytic activity of the 26S proteasome complex. Then the cell lysate extracted from control and TRIM55 overexpressing HCC cells was co-immunoprecipitated with an anti-NF90 antibody, and the ubiquitination level of NF90 was tested using an anti-Ubiquitin antibody (Cell Signaling).

HEK-293T cells were transiently transfected with various HA-tagged ubiquitin, NF90-FLAG, and TRIM55-GFP for a duration of 48 h. Following an 8-h incubation with 10 μM MG132 (MCE), the cells were collected and lysed in a denaturing lysis buffer. The HA-ubiquitinated NF90-FLAG was immunoprecipitated using the anti-FLAG M2 affinity gel (Sigma-Aldrich). The NF90-FLAG protein was purified and subjected to immunoblotting using an HA antibody (Cell Signaling).

### Animal studies

Aimal experiments were performed in which 2 × 10^6^ cells were subcutaneously injected into the right flank of 4-week-old male BALB/c athymic nude mice. Mice were randomly divided into two groups. The tumor volume was measured every 3 days and evaluated as follows: (length × width^2^)/2 mm^3^. After 19 days, the nude mice were sacrificed and tumor weights were measured for further analysis. To test the effect of TRIM55 on metastasis, 1.5 × 10^6^ cells were injected into tail vein of nude mice. After 8 weeks, the mice were sacrificed. The lung tissues were isolated and used for H&E staining according to the standard procedure. Animal work was performed using an approved Institutional Animal Care and Use Protocol approved by Xiamen University.

### RNA immunoprecipitation (RIP) assay

RIP assay was conducted using the EZ-Magna RIP Kit (Millipore) in accordance with the provided guidelines. In brief, a total of 1 × 10^7^ cells were lysed in RIP lysis buffer. The supernatant was then incubated overnight at 4 °C with magnetic beads conjugated to the 5 μg anti-NF90 antibody. Subsequently, the complexes were washed with RIP wash buffer. Finally, the immunoprecipitates were subjected to incubation with Proteinase K at 55 °C for 30 min. Following RNA extraction using Trizol regent (Invitrogen), qRT-PCR was performed to detect the levels of HIF1α or TGF-β2 mRNA.

### RNA stability assay

To assess the degradation of HIF1α, VEGF, and TGF-β2 mRNA, the administration of actinomycin D (5 mg/mL) was employed to impede the synthesis of new RNA. Following the indicated incubation period, total RNA was extracted and the remaining transcripts of HIF1α, VEGF, and TGF-β2 were quantified using qRT-PCR. The stability of HIF1α, VEGF, and TGF-β2 transcripts over time was evaluated in relation to the initial time point and standardized against that of 18S rRNA (a byproduct of RNA polymerase I, unaffected by actinomycin D).

### Tube formation assay

HUVECs at 70% confluence were subjected to overnight starvation. Metrigel High Concentration (LABLEAD, China) was carefully dispensed into pre-chilled 24-well plates (200 µl per well) and allowed to solidify for 30 min at 37 °C. The starved HUVECs were then suspended in conditioned medium (CM) obtained from stable cells and seeded onto the Matrigel at a density of 2 × 10^5^ cells per well. The plates were subsequently incubated at 37 °C. The formation of capillary-like structures was observed and recorded using a microscope, starting from 6 h after cell seeding. The quantification of tubule crossings was performed using Image-J software (Media Cybernetics, USA).

### Immunofluorescence (IF)

Cells were grown on chamber slides, fixed with 4% paraformaldehyde for 30 min and permeabilized with 0.2% Triton-X. After 3 washing with PBS, cells were incubated with an anti-NF90 (Abclonal) antibody. Secondary antibodies used were goat anti-rabbit conjugated with Alexa Fluor^TM^ 594 goat anti-rabbit IgG(H + L) (Invitrogen). The nucleus was stained with 4′,6-diamino-2-phenylindole (DAPI) before imaging.

### Luciferase reporter assay

Cells were co-transfected with a SMAD luciferase reporter plasmid (YEASEN, China). To normalize transfection efficiency, a pRL-TK reporter containing a Renilla luciferase gene was co-transfected. After 24 h of transfection, the activities of Firefly and Renilla luciferase were measured using the dual-luciferase reporter assay system (Promega). The relative luciferase activity was determined using the Varioskan Lux detection System (Thermo Scientific) and was normalized to the Renilla luciferase activity.

### Statistical analysis

Data were analyzed using GraphPad Prism 8 software. Results are expressed as the mean ± standard deviation. Statistical comparisons between groups were performed using either Student’s t-test or multi-way analysis of variance (ANOVA). The association between TRIM55 expression and clinical characteristics of HCC patients was assessed using the Chi-squared test. Spearman’s rank correlation was utilized to explore the interrelationships among the expressions of TRIM55, NF90, HIF1α, and TGF-β2. The prognostic relevance of TRIM55 expression in HCC patients was evaluated through the Kaplan–Meier survival method and the log-rank test. *P* < 0.05 was considered statistically significant.

## Supplementary information

Supplemental Figure legends and Figure

## Data Availability

The data support the findings of this study are available from the corresponding authors upon reasonable request.
