# Supplementary material for: TRIM55 restricts the progression of hepatocellular carcinoma through ubiquitin-proteasome-mediated degradation of NF90
Source: Cell Death Discov. 2024 Oct 17;10:441. doi: 10.1038/s41420-024-02212-y (PMC11487063; doi:10.1038/s41420-024-02212-y)

Supplemental Figure legends

Supplemental Figure 1. TRIM55 associates with NF90.

A. Kaplan–Meier Plotter tool was used to analyze overall survival rates of HCC patients with high and low TRIM63 expression.

B. Kaplan–Meier Plotter tool was used to analyze overall survival rates of HCC patients with high and low TRIM55 expression.

C. The results of qRT-PCR assay detecting the NF90 mRNA expression in Huh7 and SK-Hep-1 cells with TRIM55 overexpression. ns, non-significant.

Supplemental Figure 2. Knockdown of TRIM55 promotes NF90 expression.

A. Endogenous co-IP experiments indicated that TRIM55 interacted with NF90 in PLC/PRF/5 cells. IgG was used as a negative antibody.

B. NF90 expression levels in control and TRIM55 knockdown PLC/PRF/5 cells were determined via western blotting.

C. Control and TRIM55 knockdown PLC/PRF/5 were treated with CHX (200 μg/ml) for time intervals indicated. The cell lysates were subjected to immunoblotting (left panel) and NF90 expression level was quantified (right panel).

D. Control and TRIM55 knockdown PLC/PRF/5 cells were treated with MG132 (10 μM) for 8 h. Cell lysates were then subjected to immunoprecipitation with an anti-NF90 antibody. The resulting immunocomplexes were immunoblotted with anti-NF90 and anti-ubiquitin antibodies.

Supplemental Figure 3. The downstream signaling regulated by TRIM55.

A. The Gene Ontology (GO) analysis on the genes significantly affected by TRIM55.

B. The Kyoto Encyclopedia of Genes and Genomes (KEGG) analysis on the genes significantly affected by TRIM55.

Supplemental Figure 4. TRIM55 regulates HIF1α and TGF-β2.

A. Correlation analysis between NF90 and HIF1α in HCC samples from TCGA database.

B. Representative images of IHC staining of HIF1α in HCC tissues with different expression levels of NF90. Scatter diagram exhibited a positive correlation of NF90 and HIF1α in HCC tissues by IHC.

C. Correlation analysis between NF90 and TGF-β2 in HCC samples from TCGA database.

D. Representative images of IHC staining of TGF-β2 in HCC tissues with different expression levels of NF90. Scatter diagram exhibited a positive correlation of NF90 and TGF-β2 in HCC tissues by IHC.

Supplemental Figure 5. TRIM55 inhibits HCC progression partially through HIF1α and TGF-β2.

A. HIF1α was transfected into in Huh7 and SK-Hep-1 cells with TRIM55 overexpression.

B. Huh7 and SK-Hep-1 cells with TRIM55 overexpression were treated with TGF-β2.

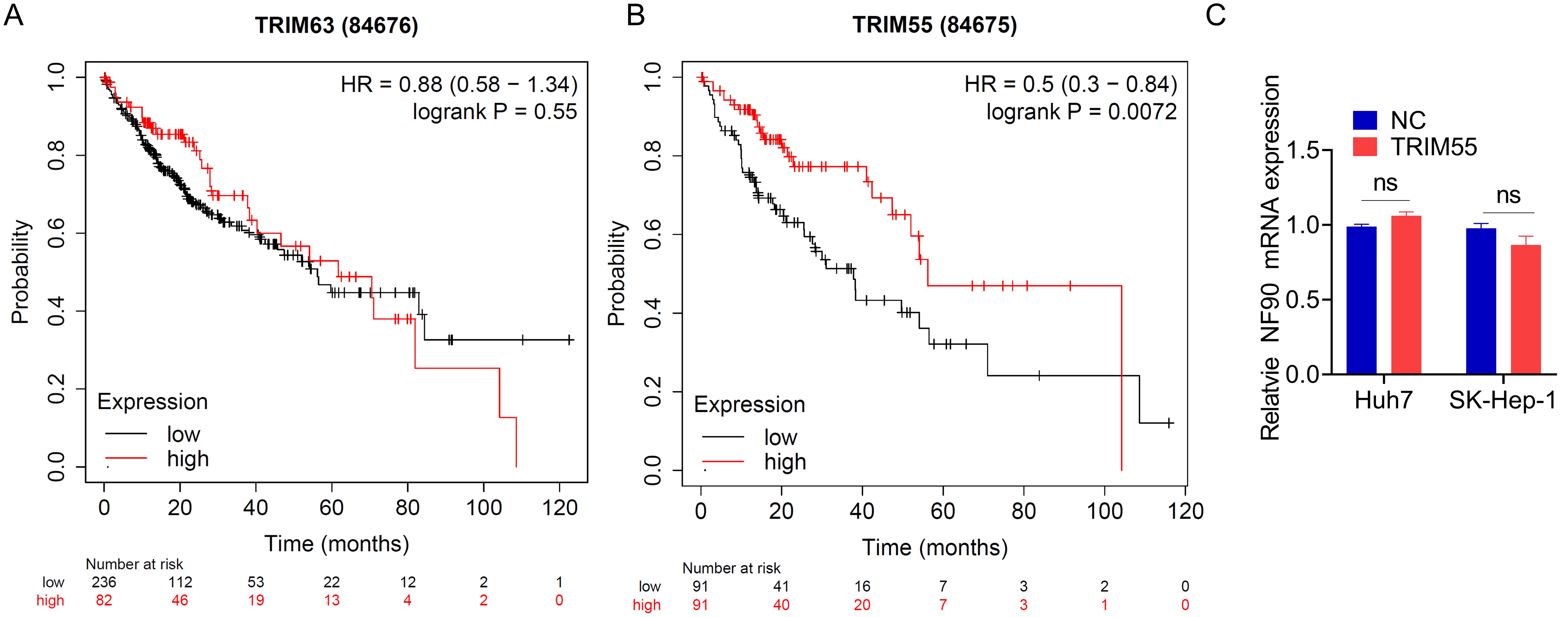
A
TRIM63 (84676)
HR = 0.88 (0.58 − 1.34)
logrank P = 0.55
Probability
Time (months)
Expression
low
high
Number at risk
low 236 112 53 22 12 2 1
high 82 46 19 13 4 2 0
B
TRIM55 (84675)
HR = 0.5 (0.3 − 0.84)
logrank P = 0.0072
Probability
Time (months)
Expression
low
high
Number at risk
low 91 41 16 7 3 2 0
high 91 40 20 7 3 1 0
C
NC
TRIM55
ns
ns
Relatvie NF90 mRNA expression
Huh7
SK-Hep-1

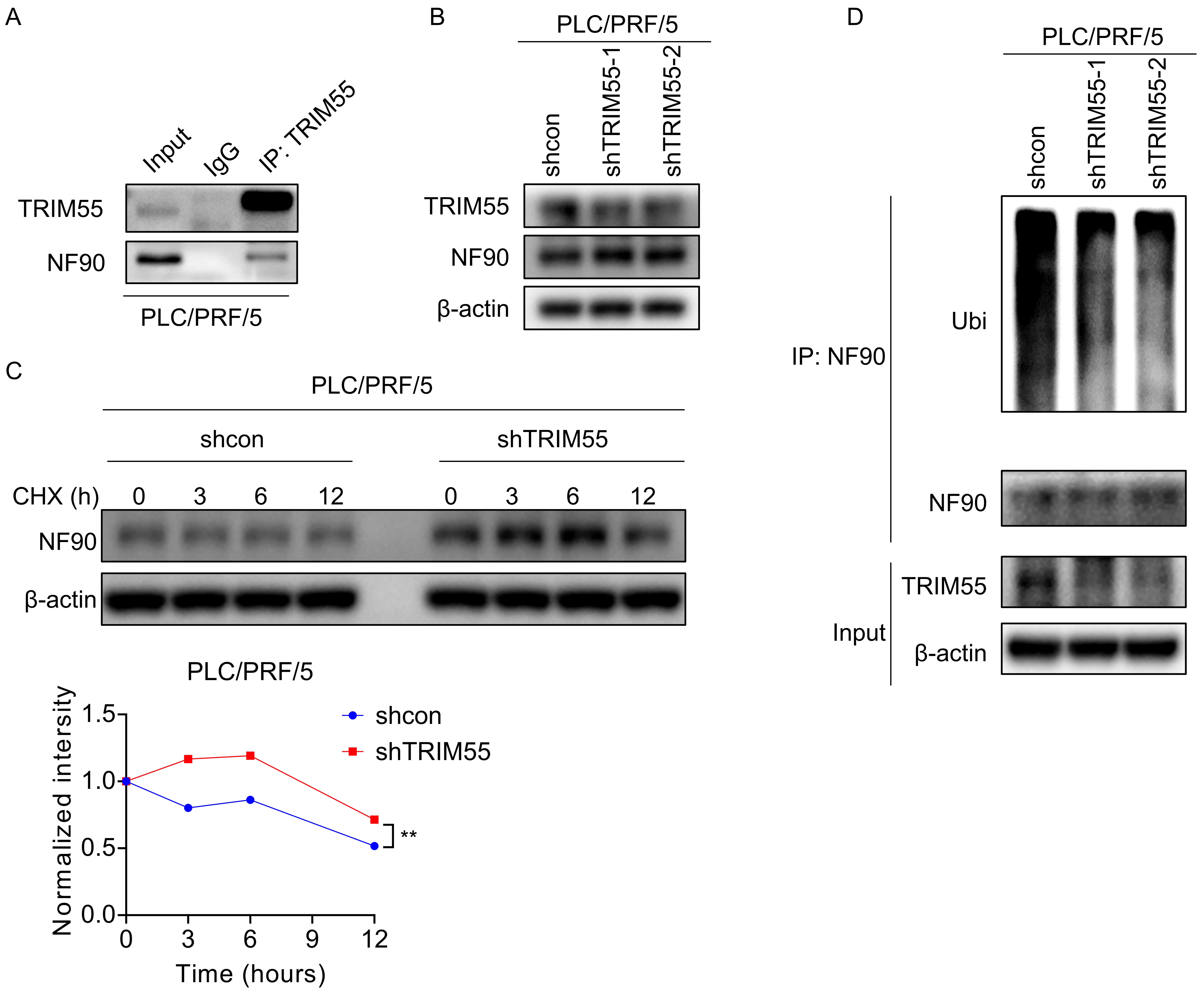
A
Input
IgG
IP: TRIM55
TRIM55
NF90
PLC/PRF/5
B
PLC/PRF/5
shcon
shTRIM55-1
shTRIM55-2
TRIM55
NF90
β-actin
C
PLC/PRF/5
shcon
shTRIM55
CHX (h)
0
3
6
12
NF90
β-actin
PLC/PRF/5
shcon
shTRIM55
Normalized intersity
Time (hours)
**
D
PLC/PRF/5
shcon
shTRIM55-1
shTRIM55-2
IP: NF90
Ubi
NF90
Input
TRIM55
β-actin

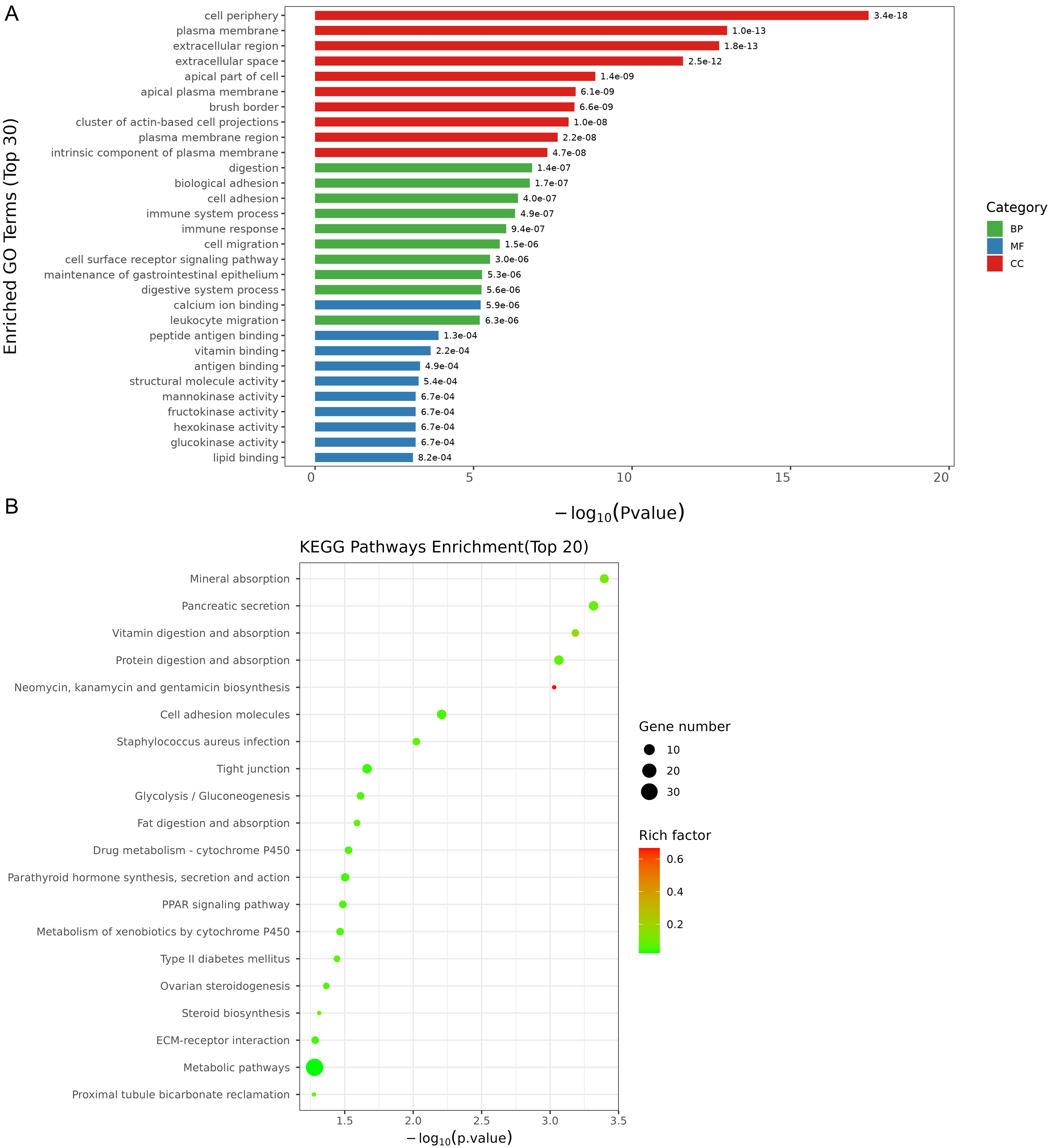
A
Enriched GO Terms (Top 30)
cell periphery
plasma membrane
extracellular region
extracellular space
apical part of cell
apical plasma membrane
brush border
cluster of actin-based cell projections
plasma membrane region
intrinsic component of plasma membrane
digestion
biological adhesion
cell adhesion
immune system process
immune response
cell migration
cell surface receptor signaling pathway
maintenance of gastrointestinal epithelium
digestive system process
calcium ion binding
leukocyte migration
peptide antigen binding
vitamin binding
antigen binding
structural molecule activity
mannokinase activity
fructokinase activity
hexokinase activity
glucokinase activity
lipid binding
3.4e-18
1.0e-13
1.8e-13
2.5e-12
1.4e-09
6.1e-09
6.6e-09
1.0e-08
2.2e-08
4.7e-08
1.4e-07
1.7e-07
4.0e-07
4.9e-07
9.4e-07
1.5e-06
3.0e-06
5.3e-06
5.6e-06
5.9e-06
6.3e-06
1.3e-04
2.2e-04
4.9e-04
5.4e-04
6.7e-04
6.7e-04
6.7e-04
6.7e-04
8.2e-04
0
5
10
15
20
$-\log_{10}(\text{Pvalue})$
Category
BP
MF
CC
B
KEGG Pathways Enrichment(Top 20)
Mineral absorption
Pancreatic secretion
Vitamin digestion and absorption
Protein digestion and absorption
Neomycin, kanamycin and gentamicin biosynthesis
Cell adhesion molecules
Staphylococcus aureus infection
Tight junction
Glycolysis / Gluconeogenesis
Fat digestion and absorption
Drug metabolism - cytochrome P450
Parathyroid hormone synthesis, secretion and action
PPAR signaling pathway
Metabolism of xenobiotics by cytochrome P450
Type II diabetes mellitus
Ovarian steroidogenesis
Steroid biosynthesis
ECM-receptor interaction
Metabolic pathways
Proximal tubule bicarbonate reclamation
1.5
2.0
2.5
3.0
3.5
$-\log_{10}(\text{p.value})$
Gene number
10
20
30
Rich factor
0.6
0.4
0.2

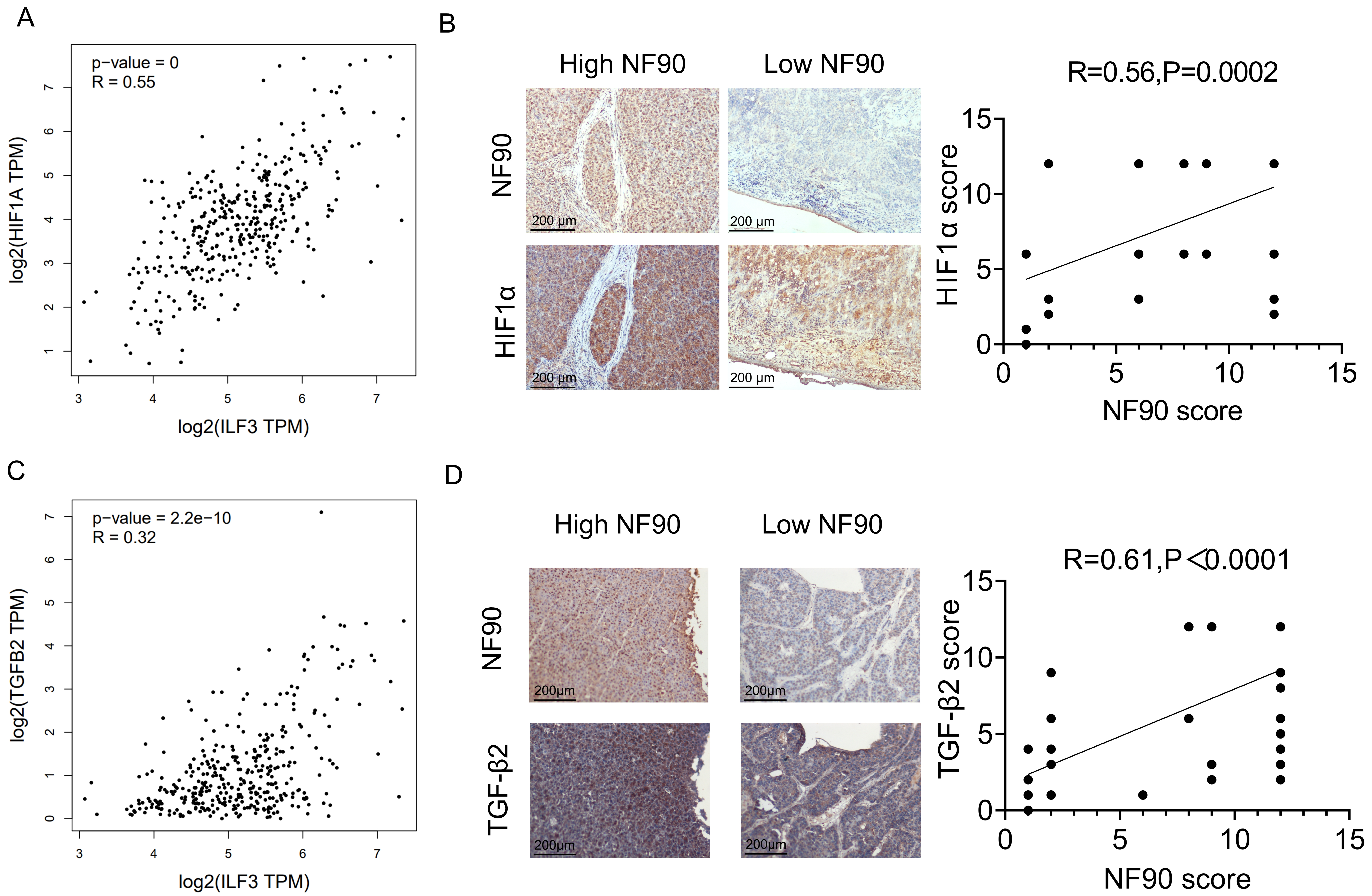
A
p−value = 0
R = 0.55
log2(HIF1A TPM)
log2(ILF3 TPM)
B
High NF90
Low NF90
NF90
HIF1α
200 μm
R=0.56,P=0.0002
HIF1α score
NF90 score
C
p−value = 2.2e−10
R = 0.32
log2(TGFB2 TPM)
log2(ILF3 TPM)
D
High NF90
Low NF90
NF90
TGF-β2
200μm
R=0.61,P<0.0001
TGF-β2 score
NF90 score

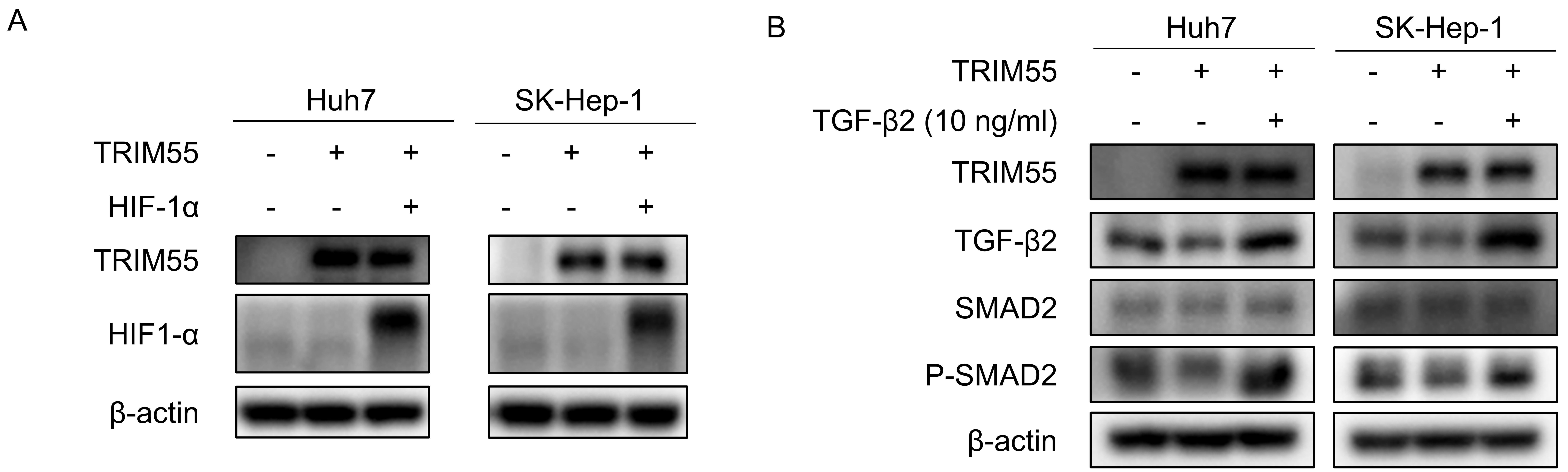
A
Huh7
SK-Hep-1
TRIM55 - + + - + +
HIF-1α - - + - - +
TRIM55
HIF1-α
β-actin
B
Huh7
SK-Hep-1
TRIM55 - + + - + +
TGF-β2 (10 ng/ml) - - + - - +
TRIM55
TGF-β2
SMAD2
P-SMAD2
β-actin

Figure 2

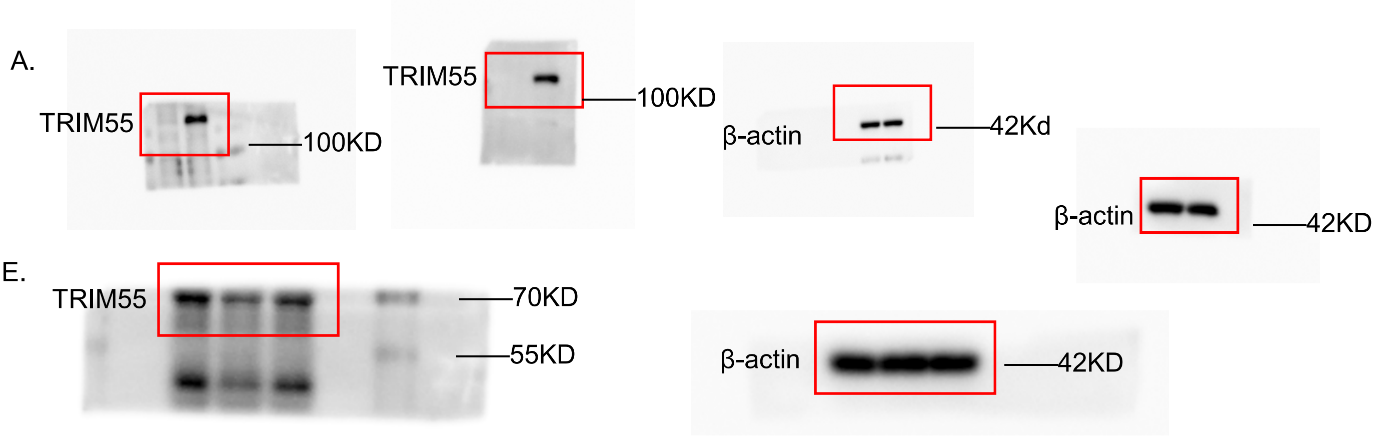

Figure 5

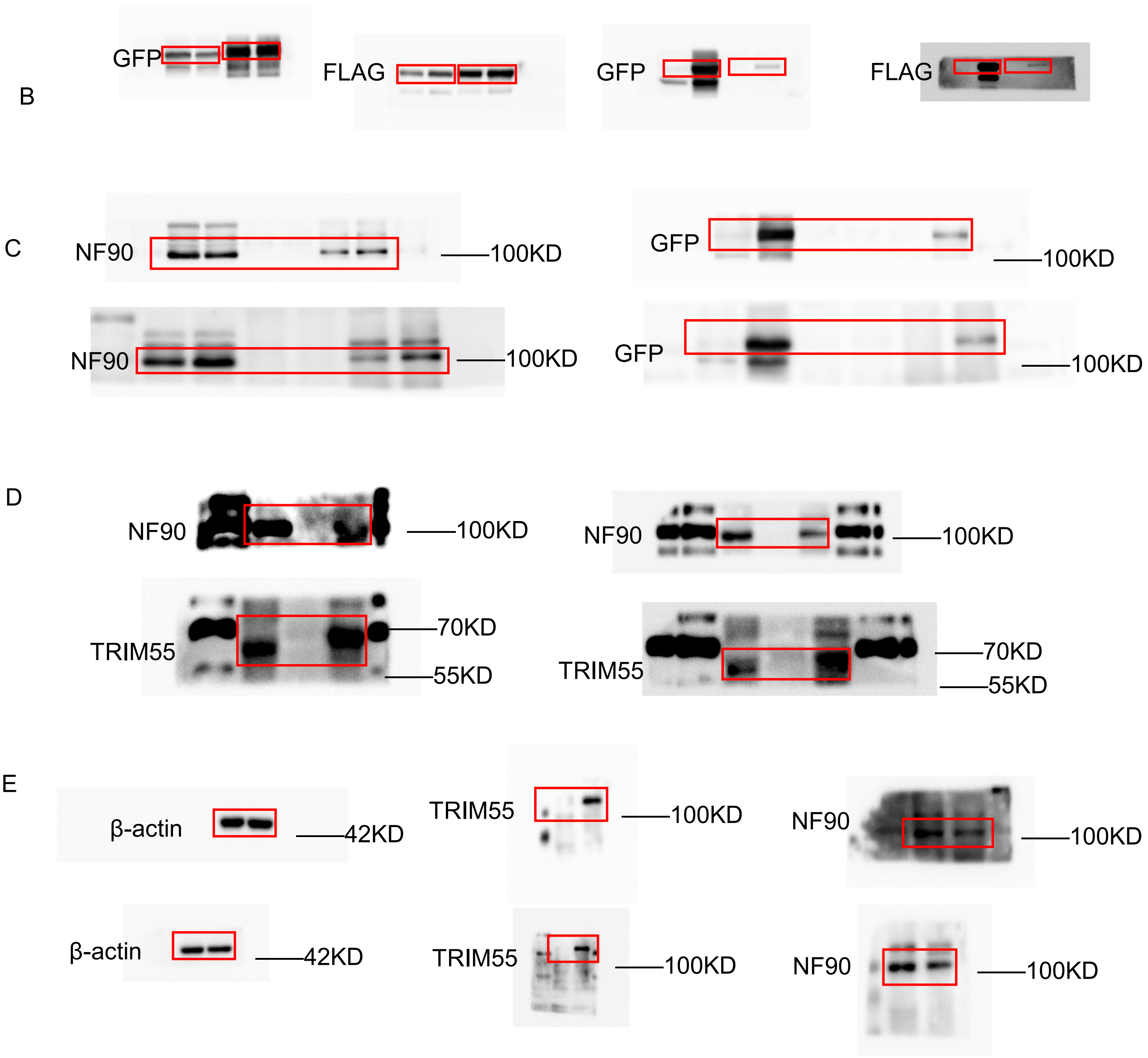

Figure 5

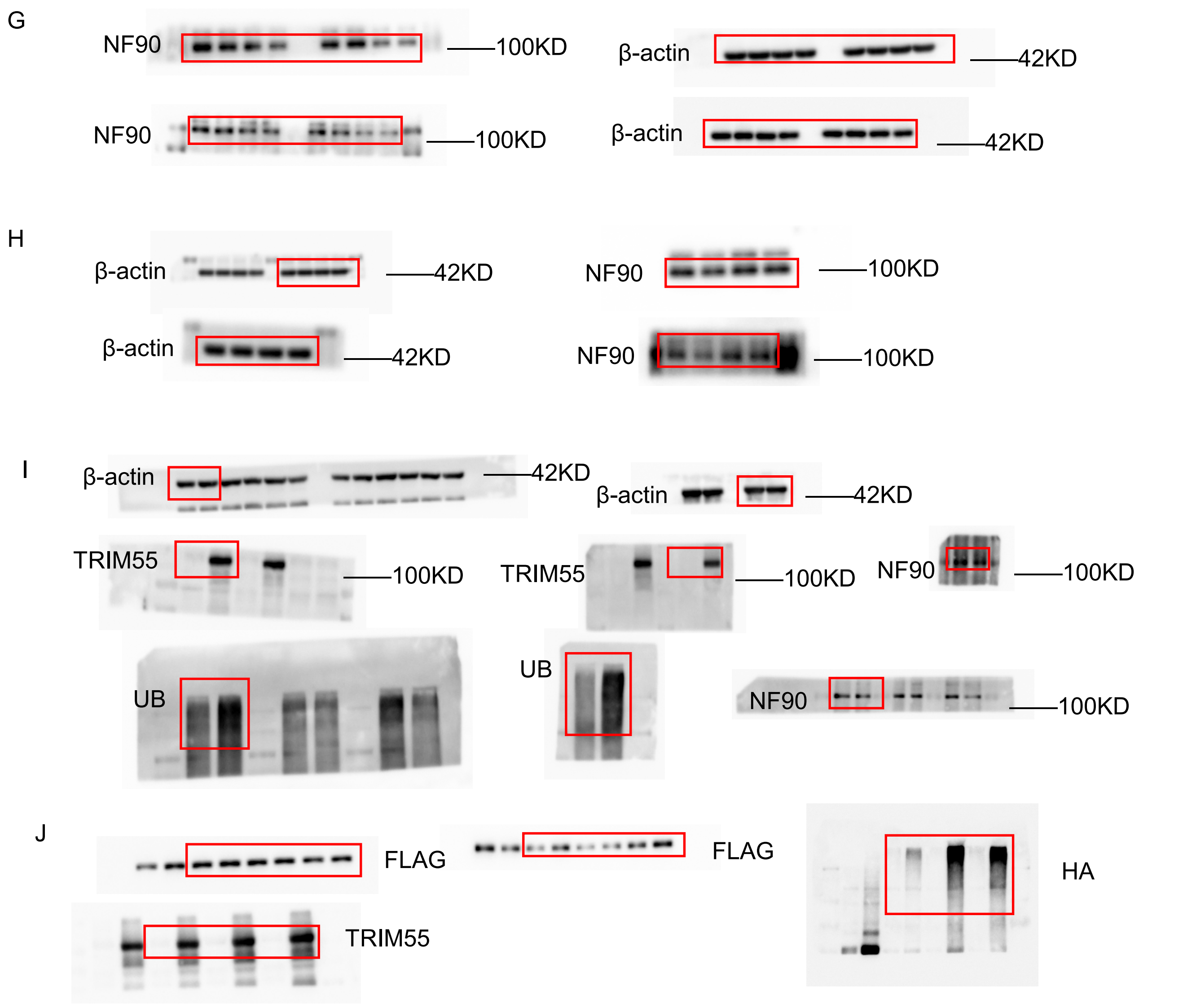

Figure 6

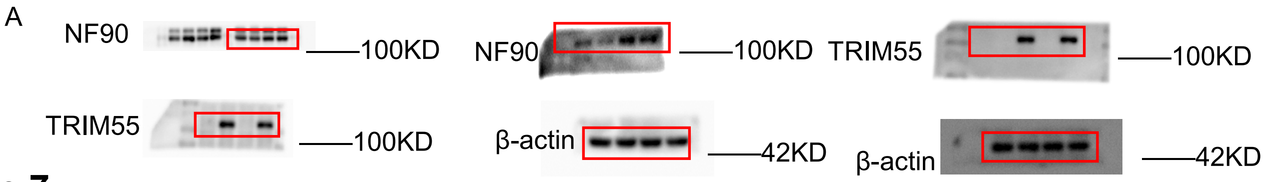

Figure 7

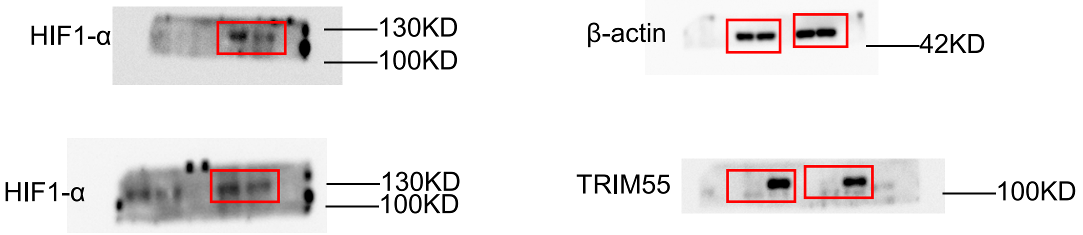

Figure 8

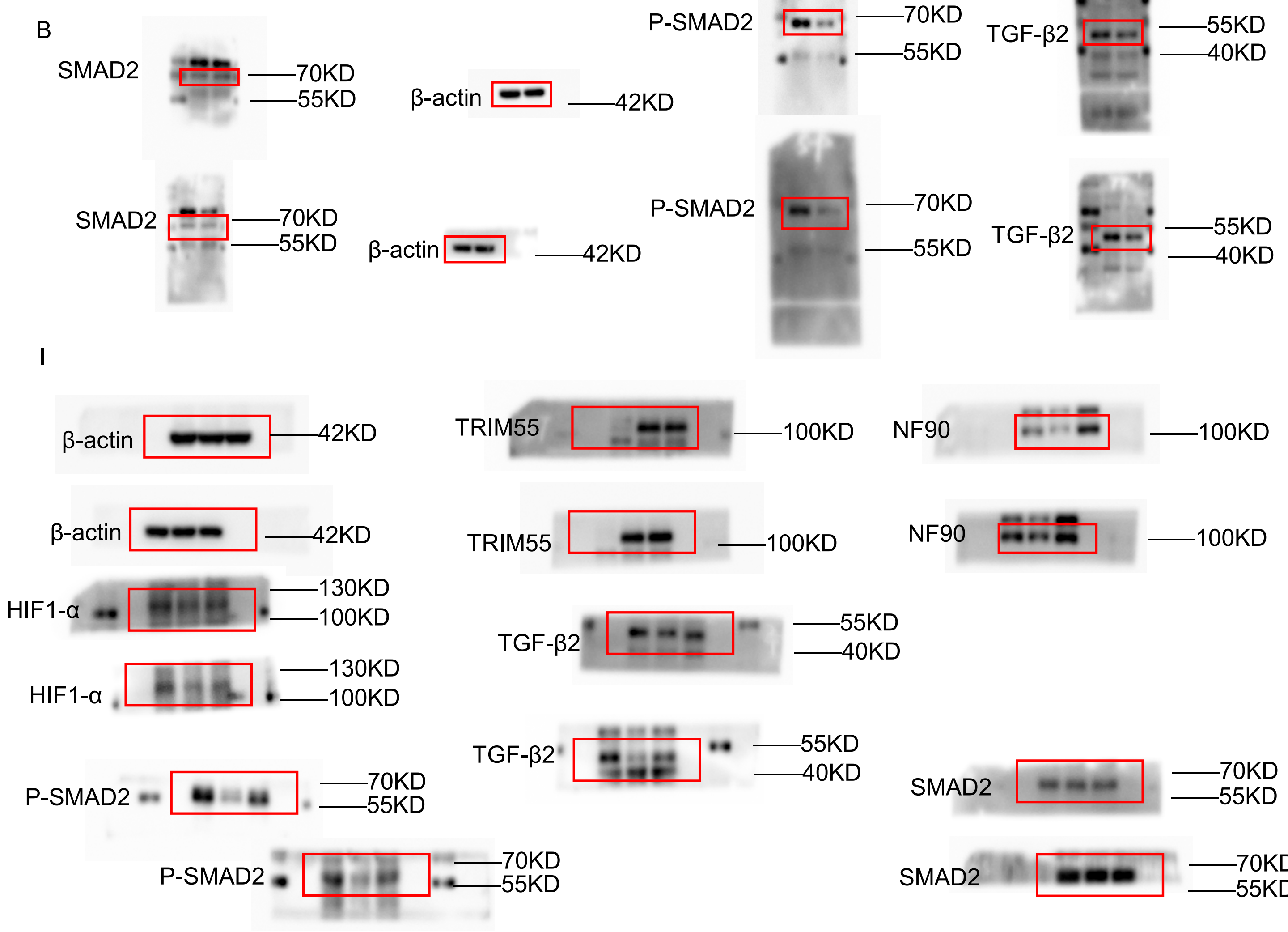

Supplemental Figure 2

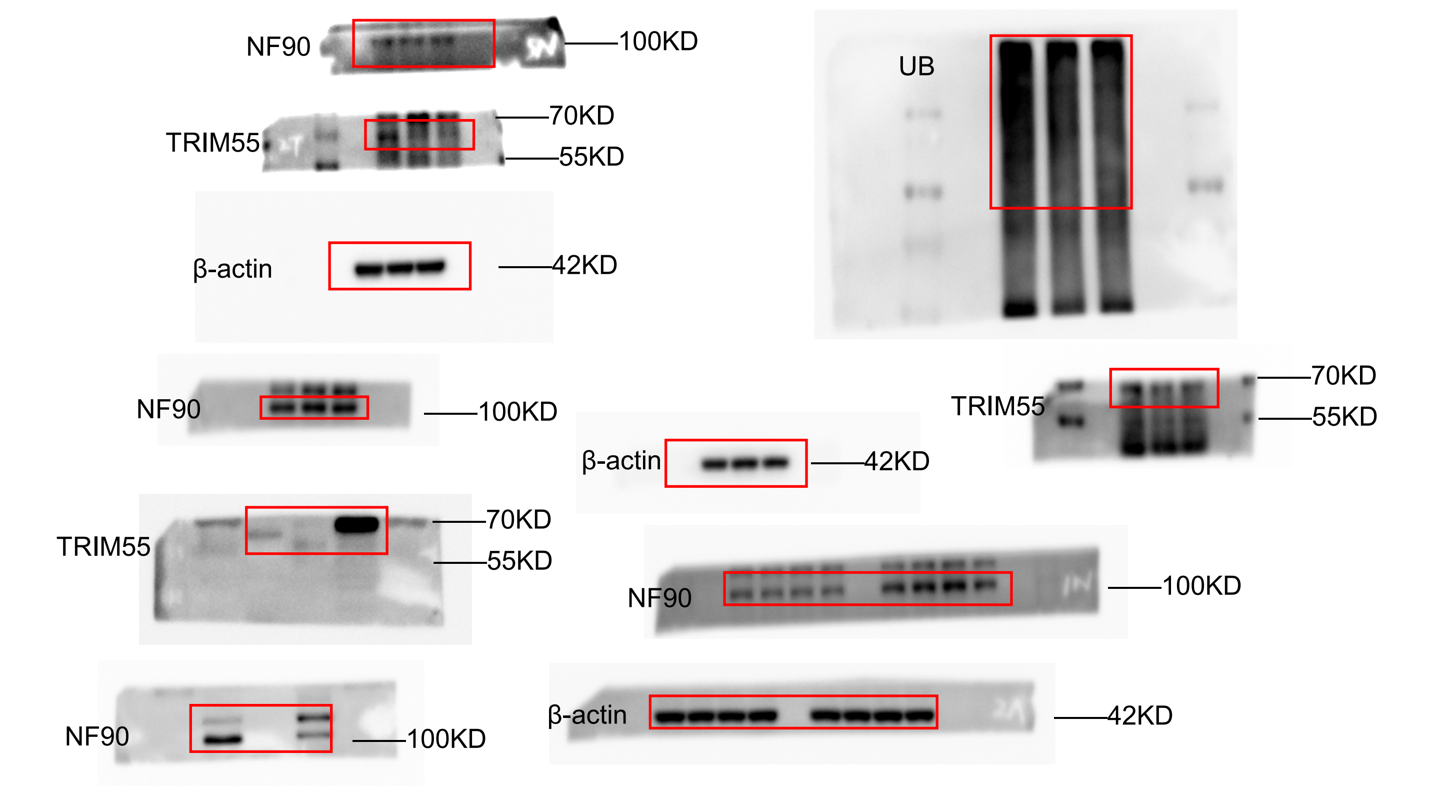

Supplemental Figure 5

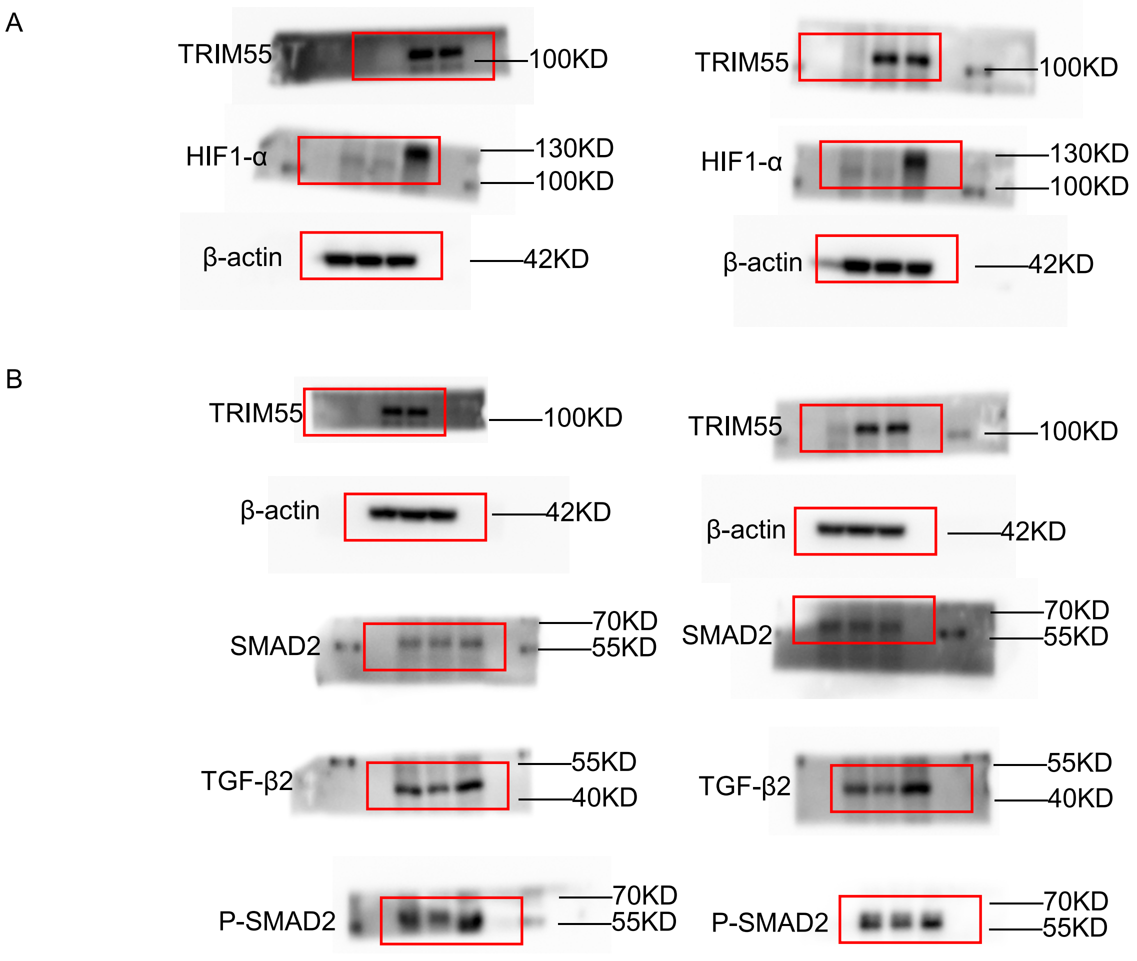

Supplement: Supplementary file 1 — Supplemental Figure legends and Figure [file 41420_2024_2212_MOESM1_ESM.pdf]
